# Effects of Systemically Administered Hydrocortisone on the Human Immunome

**DOI:** 10.1038/srep23002

**Published:** 2016-03-14

**Authors:** Matthew J. Olnes, Yuri Kotliarov, Angélique Biancotto, Foo Cheung, Jinguo Chen, Rongye Shi, Huizhi Zhou, Ena Wang, John S. Tsang, Robert Nussenblatt, The CHI Consortium, Howard B. Dickler, Christopher S. Hourigan, Francesco M. Marincola, J. Phillip McCoy, Shira Perl, Paula Schum, Pamela L. Schwartzberg, Giorgio Trinchieri, Janet Valdez, Neal S. Young

**Affiliations:** 1 Trans-NIH Center for Human Immunology, Autoimmunity, and Inflammation (CHI), National Institutes of Health (NIH), Bethesda, MD, 20892, USA; 2 Hematology Branch, National Heart, Lung, and Blood Institute (NHLBI), Bethesda, MD, 20892, USA; 3 Systems Genomics and Bioinformatics Unit, Laboratory of Systems Biology, National Institute of Allergy and Infectious Diseases, NIH, Bethesda, MD, 20892, USA; 4 Laboratory of Immunology, National Eye Institute, NIH, Bethesda, MD, 20892, USA; 5 Myeloid Malignancies Section, NHLBI, NIH, Bethesda, MD, 20892 USA; 6 Genetic Disease Research Branch, National Human Genome Research Institute, NIH, Bethesda, MD, 20892 USA; 7 Program in Cancer and Inflammation, National Cancer Institute, NIH, Bethesda, MD, 20892, USA

## Abstract

Corticosteroids have been used for decades to modulate inflammation therapeutically, yet there is a paucity of data on their effects in humans. We examined the changes in cellular and molecular immune system parameters, or “immunome”, in healthy humans after systemic corticosteroid administration. We used multiplexed techniques to query the immunome in 20 volunteers at baseline, and after intravenous hydrocortisone (HC) administered at moderate (250 mg) and low (50 mg) doses, to provide insight into how corticosteroids exert their effects. We performed comprehensive phenotyping of 120 lymphocyte subsets by high dimensional flow cytometry, and observed a decline in circulating specific B and T cell subsets, which reached their nadir 4–8 hours after administration of HC. However, B and T cells rebounded above baseline 24 hours after HC infusion, while NK cell numbers remained stable. Whole transcriptome profiling revealed down regulation of NF-κB signaling, apoptosis, and cell death signaling transcripts that preceded lymphocyte population changes, with activation of NK cell and glucocorticoid receptor signaling transcripts. Our study is the first to systematically characterize the effects of corticosteroids on the human immunome, and we demonstrate that HC exerts differential effects on B and T lymphocytes and natural killer cells in humans.

Glucocorticoids were recognized to modulate the immune system as early as 1924, when it was observed that adrenalectomy caused thymic hyperplasia in rats^1^. Cortisone acetate and adrenocorticotropic hormone were first used in 1948 to treat rheumatoid arthritis^2^, and corticosteroids have since become the most commonly prescribed class of immune modulatory therapeutics. Despite the widespread use of glucocorticoids in clinical medicine, their effects on broad aspects of the cellular and molecular immune response, or the human “immunome”^3^ have not been examined in detail.

Numerous studies have evaluated the effects of corticosteroids on immune parameters in animals, and in human cells cultured *in vitro*^4^,^5^,^6^,^7^. In landmark work, Dale and colleagues demonstrated that hydrocortisone administered intravenously to healthy volunteers at doses as high as 400 mg for two consecutive days was well-tolerated and caused rapid mobilization of neutrophils^8^; 400 mg hydrocortisone transiently decreased recirculating concanavalin-A responsive peripheral (T) cells, while 100 mg of hydrocortisone did not induce lymphopenia in humans^9^,^10^. In other studies, glucocorticoids reduced autologous mixed lymphocyte responses^11^, depleted CD14+CD16+ monocytes, inhibited IL-10 and CD163 expression in monocytes^12^, decreased levels of TNFα and soluble TNFα receptor^13^, and diminished numbers of plasmacytoid dendritic cells in normal volunteers^14^. These studies were conducted prior to the advent of modern multiplexed methods.

Given the activity of the ligand-engaged glucocorticoid receptor (GR) as DNA binding protein, changes in gene expression are an expected outcome of GC administration, but how and to what extent glucocorticoids exert their immunosuppressive and anti-inflammatory effects through such gene expression changes in humans is not well understood. While effects of corticosteroids on gene expression in animals and cultured human PBMCs have been examined^15^,^16^,^17^, to our knowledge, there is no published investigation of the global effects on gene expression in response to systemically administered glucocorticoids in healthy subjects.

## Results

### Effects of systemically administered HC on human lymphocytes

**Figure 1:**
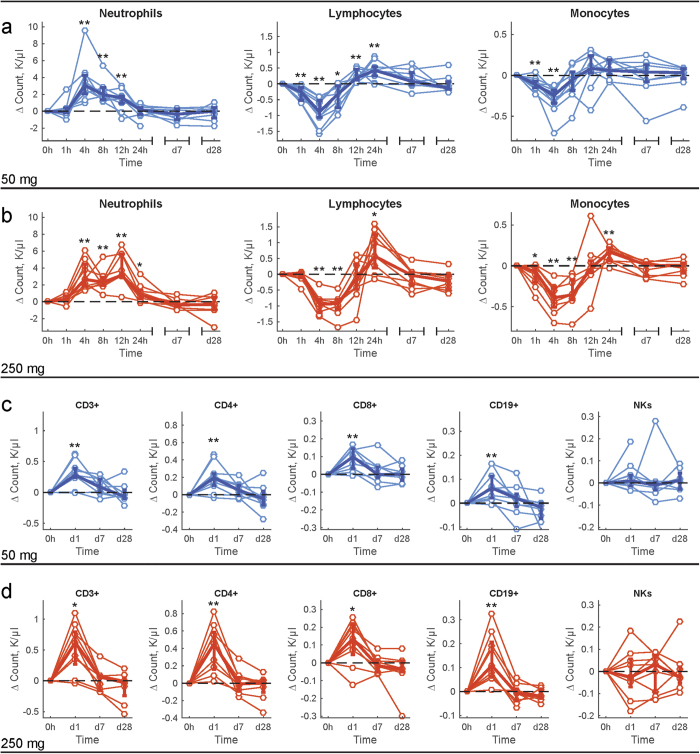
Effects of systemically administered hydrocortisone (HC) on circulating peripheral blood cells in humans. Healthy volunteers had phlebotomy at baseline and after they were administered 50 mg (**a,c**) or 250 mg (**b,d**) intravenous HC. (**a,b**): absolute neutrophil counts, absolute lymphocyte counts, and absolute monocyte counts were determined by complete blood counts. (**c,d**): Quantitation of CD45+CD3+ T cells, CD45+CD3+CD8-CD4+ T cells, CD45+CD3+CD4-CD8+ T cells, CD45+CD3-CD19+ B cells, and CD45+CD3-CD16+CD56+ NK cells were determined by clinical flow cytometry as described in the Materials and Methods. Each panel depicts data from up to ten individuals treated with either 50 mg or 250 mg HC.Data for a single patient are shown as a single thin line. Think line represents median across patients with error bars indicating 25-th and 75-th quantile. Time points with significant changes in cell counts comparing with baseline are marked with *for *p* < 0.05, and **for *p* < 0.01 (Wilcoxon signed-rank test).

High doses of HC (400 mg) cause transient neutrophil demargination and a decrease in circulating lymphocytes, effects that peak at four hours^8^, while 100 mg of HC does not induce lymphocyte depletion in humans^9^,^10^. To further characterize the effects of corticosteroids in humans, we infused 20 healthy volunteers with IV HC at 50 mg or 250 mg doses, and measured neutrophil, lymphocytes, and monocyte levels at baseline, and one hour, four hours, eight hours, 12 hours, 24 hours, seven days, and 28 days after HC infusion as described in the Materials and Methods and Fig. S1. Neutrophils increased and peaked at four hours after both 250 mg and 50 mg HC with a return to baseline levels at 24 hours (Fig. 1a,b). The magnitude of neutrophil increase did not differ between doses, while the duration of neutrophil increase was more prolonged after 250 mg HC (Fig. 1b). Lymphocytes and monocytes decreased to a similar extent after both doses of HC (Fig. 1a,b), with a nadir at four hours after 50 mg HC and cell numbers returning to baseline by 12 hours, while levels were lowest between 4–8 hours after 250 mg HC (Fig. 1b). Lymphocyte numbers increased above baseline 24 hours after both doses, with a higher magnitude rebound occurring after the 250 mg dose (p = 0.0030, Fig. 1b).

### Effects of systemically administered HC on T cells, B cells, and NK cells

Flow cytometry using reference beads was performed to determine absolute cell counts of circulating T cells, CD4+ T cells, CD8+ T cells, B cells, and NK cells. Circulating T cells levels increased 24 hours after infusion of 250 mg HC (p = 0.0024), with a return to baseline by seven days (Fig. 1c,d). CD4+ cells also increased 24 hours after 250 mg HC, as did CD19+ B lymphocytes (p = 0.0026, and p = 0.0018, respectively Fig. 1d). NK cell levels were unchanged from baseline at 24 hours after 50 mg or 250 mg HC infusion (Fig. 1c,d).

### Effects of systemically administered HC on human lymphocyte subsets

**Figure 2:**
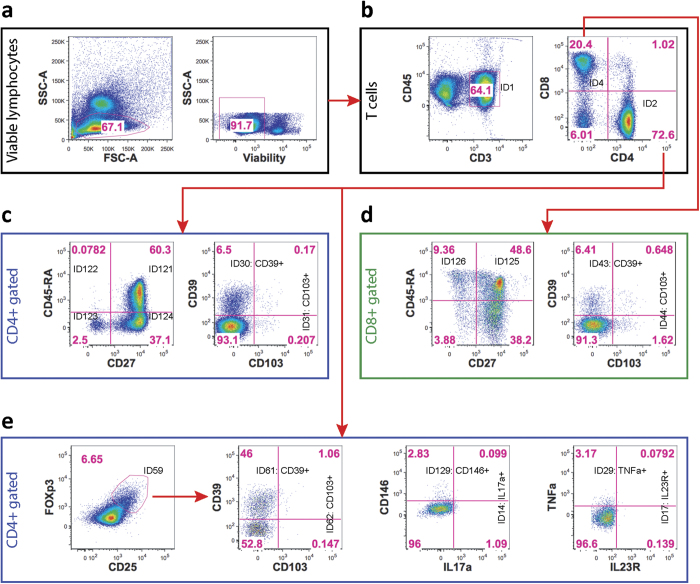
Staining patterns and gating strategies for T lineage. PBMCs were stained according to the Methods section. Debris was excluded from analysis by gating on FSC vs. SSC and (**a**) live mononuclear cells were selected by gating on the cells that excluded the viability dye. Doublets of cells were eliminated using width measurements. T cells were identified by the expression of CD45 and CD3 (**b**), and T cell subsets were identified by expression of CD4 and/or CD8. Both CD4+ and CD8+ subsets were examined for expression of surface markers allowing definition of memory and naïve cells subpopulations (CD27, CD45RA-RO, CCR7, panels **c** and **d**). Markers associated with the populations of interest (e.g. Treg, Th17, Th22) included FOXp3, CD25, CD39, CD103, CD146, and cytokines (IL17a, TNFa) staining were used to gate and characterize these cells (**e**).

**Figure 3:**
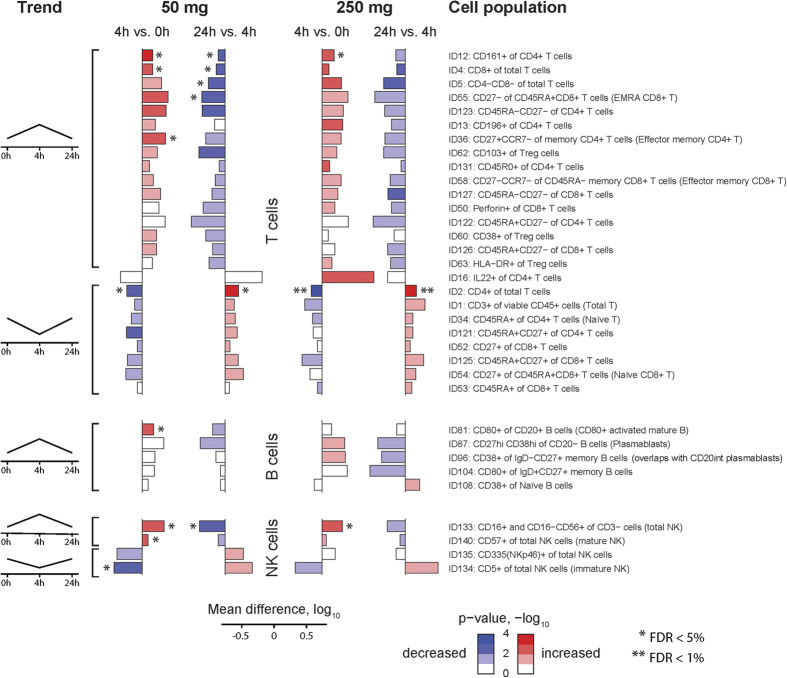
Coherently changed cell populations following HC treatment. Cell frequencies at 4 hours compared to baseline and at 24 hours comparing to four hours (pairwise t-test’s FDR corrected p-values less than 0.1 and absolute log-fold-change (of percent of parent population) higher than 0.1 for at least one comparison) for subjects receiving 50 mg (left) or 250 mg (right) of steroid. Populations are grouped by tubes (T, B and NK cells) and sorted by change in direction (indicated by trend line on the left) and meta p-value across all comparisons). Bars are colored by log10 (p-value), with blue and red colors denoting decrease or increase in percent of parent population, respectively. Asterisks are added if FDR corrected p-value is <0.05 (*) or <0.01 (**).

High dimensional comprehensive lymphocyte immune phenotyping (CLIP)^18^,^19^ was used to characterize effects of HC on B and T lymphocyte subsets and NK cells in human peripheral blood mononuclear cells (PBMCs). Healthy volunteers received 50 mg or 250 mg HC, and 120 discrete lymphocyte and NK cell subsets were analyzed by CLIP at baseline, four hours, 24 hours, and seven days (Fig. 2 and S2; Tables S1 and S2); differential effects of 50 mg and 250 mg after treatment are shown (Fig. 3, Table S3).

T cell frequencies decreased four hours after administration of 50 mg (p = 0.015) and 250 mg HC (p = 0.017), but rebounded at 24 hours (Fig. 3), consistent with the clinical flow analysis (Fig. 1). Double negative (CD4−CD8−) T cell frequencies were decreased four hours after both HC doses, while CD4+CD8+ cells were not changed (Table S3).

Total CD4+ T cell frequencies decreased four hours relative to baseline after both 50 mg and 250 mg HC, with a lower nadir after 250 mg HC (p = 0.00030 and p = 0.00016, respectively). However, among the CD4+ T cell subsets, effector T helper cell frequencies were increased four hours after 50 mg (p = 0.0092) and 250 mg (p = 0.047) HC. Effector memory cell frequencies were increased four hours after 50 mg HC administration (p = 0.0039), although this did not reach significance after 250 mg HC (p = 0.057). Memory helper T cell frequencies were also increased four hours after both doses of HC (p = 0.036, and p = 0.0073). In contrast, naïve helper T cell frequencies were decreased four hours after both doses of HC (p = 0.019, and 0.029, respectively), and CD27+ naïve T helper cell frequencies were decreased four hours after 50 mg of HC (p = 0.0093). Thus, although overall CD4+ T cell frequencies were low early, this decrease appeared primarily to affect naïve CD4+ populations.

Further analysis of CD4+ cell populations (Fig. 3, Table S3) demonstrated that certain differentiated subsets were variably affected. CD161+ Th17 cell frequencies were increased four hours after both 50 mg (p = 0.00052) and 250 mg HC (p = 0.0012), and they returned to baseline after 24 hours. CD196+ Th17 cell frequencies also increased four hours after 50 mg (p = 0.012) and 250 mg (p = 0.027) HC treatment. Total regulatory T cell frequencies (Tregs) were not changed four hours after HC administration (Table S2). Together, these observations suggest that although overall CD4+ T cell frequencies were decreased, the effects on specific cell populations differed.

In contrast to the effects on CD4+ T cells, total CD8+ T cell frequencies were increased four hours after 50 mg (p = 0.0022) and 250 mg (p = 0.0073) HC (Fig. 3). Naïve CD8+ T cells frequencies were decreased four hours after 50 mg of HC (p = 0.0028), whereas effector memory CD8+ T cells were increased four hours after 50 mg HC (p = 0.021), but not 250 mg (p = 0.085). Cytotoxic T effector memory RA+ (EMRA) cell frequencies were increased four hours after both doses of HC (p = 0.0072, and 0.024). Activated cytotoxic CD8+ T cell frequencies were not changed after HC (Table S3). Thus, CD8+ T cell subsets also exhibited differential responses to systemic HC administration, with an increased frequency of total CD8+ T cells.

B cell subsets exhibited fewer changes than T cells four hours after HC administration, with frequencies of total B cells showing no changes (Table S3). Among the B cell subsets (Fig. 3), mature activated B cell frequencies were increased four hours after 50 mg HC (p = 0.0047), while the 250 mg dose increased frequencies of plasmablasts (p = 0.052). Total NK cell frequencies were increased four hours after both 50 mg and 250 mg HC (p = 0.0061, p = 0.0030). Steroids exerted a differential effect on frequencies of NK subsets, with mature NK cells increasing four hours after both doses of HC (p = 0.0027, p = 0.046), and immature NK cells decreasing after both doses (p = 0.0060, p = 0.031) of HC (Fig. 3). Virtually all T and NK cell populations that exhibited frequency changes at four hours returned to baseline values at 24 hours (Fig. S2).

### Effects of systemically administered HC on gene expression in human PBMCs

**Figure 4:**
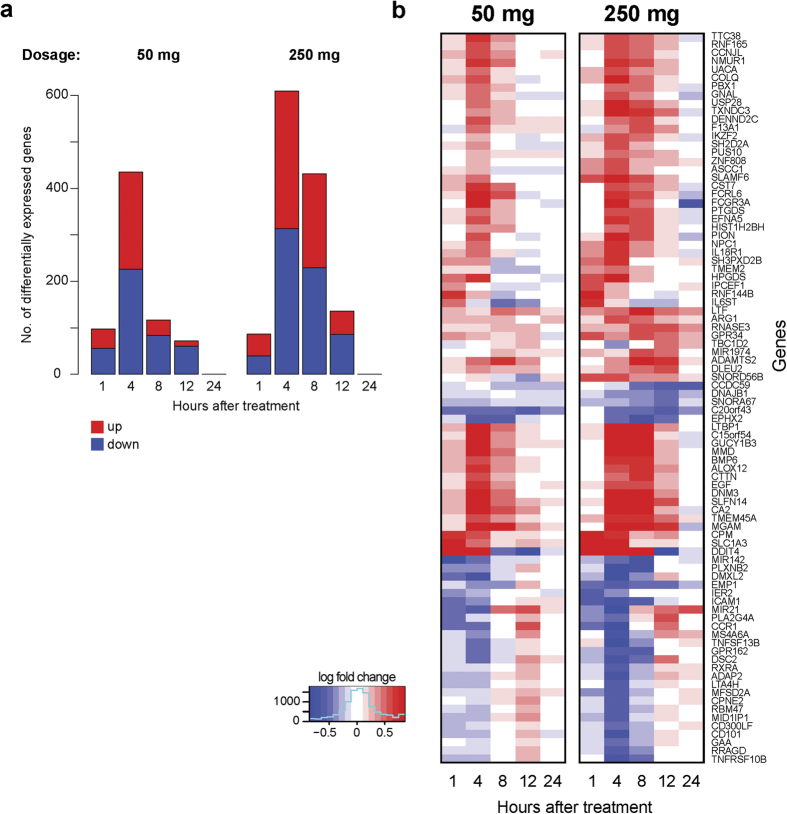
Post-treatment coherent changes in gene transcription. (**a**) Number of differentially expressed genes (FDR <0.05 and absolute log-fold-change >0.5) for subjects received 50 mg (left) or 250 mg (right) of HC between one and 24 hours comparing to baseline (0 hour). (**b**) Top differentially expressed genes (88) with FDR <0.05 and absolute log-fold-change >1 at least for one comparison.

We performed whole transcriptome gene expression microarray studies using RNA and analysis of 21,608 transcripts. The 50 mg and 250 mg doses of HC exert qualitatively different effects on gene expression with distinct kinetics. Changes in gene expression were observed as early as one hour after HC infusion, with peak changes in gene expression occurring after four hours, and more changes observed with the 250 mg dose (Fig. 4). Progressively fewer changes in gene expression were observed four hours after HC administration. We did not observe any statistically significant differences between males and females in HC induced gene expression (data not shown).

**Figure 5:**
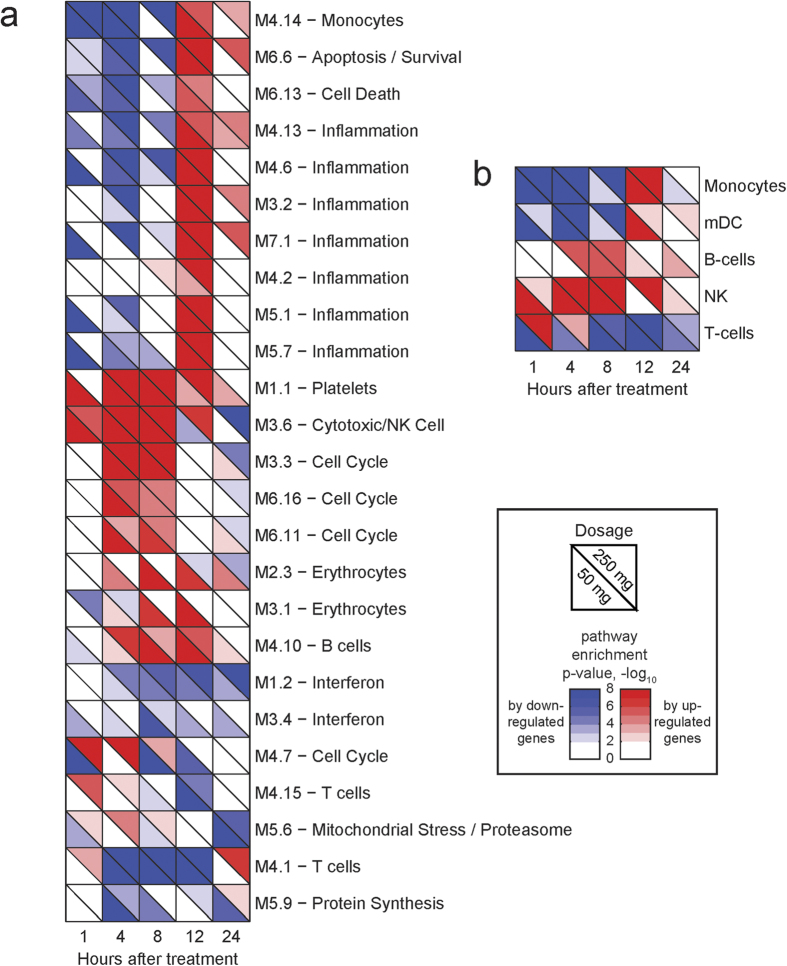
Blood transcriptome modules^20^ and cell specific gene sets 21 enriched in coherently changing genes after systemic administration of 50 mg and 250 mg of HC. The enrichment analysis was performed separately for up-regulated (enrichment p-values are represented by red colors) and down-regulated (blue colors) genes. The Transcription modules (**a**) and cell population specific gene sets (**b**) with FDR corrected enrichment p-values less than 1 × 10^−5^ at least at one time points and one HC dosage were included. For each module/timepoint cell color in lower left corner illustrates p-value for 50 mg dosage, and in upper right corner – for 250 mg.

**Figure 6:**
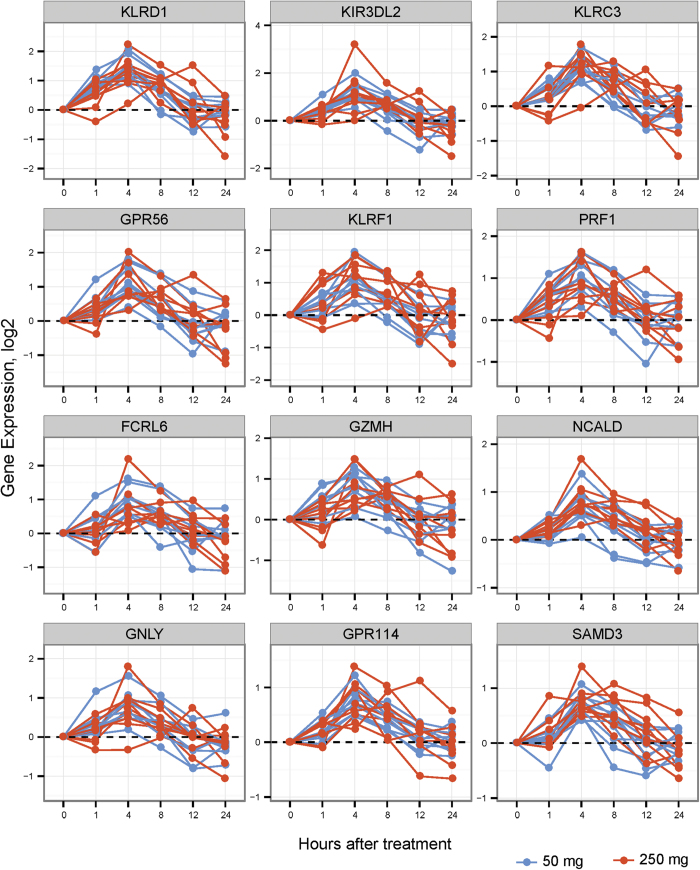
Natural killer cell gene signature changes after HC administration. Gene expression profiles of top genes from the Cytotoxic/NK Cell blood transcriptome module (labeled M3.6 on Fig. 5a) with largest change from baseline to 4 hours. Each line represents an individual patient with blue and red color indicating patients receiving 50 or 250 mg of hydrocortisone respectively.

To place these gene expression changes in a broader context, we next performed gene set enrichment analysis of mRNAs significantly changed from baseline at different time points after both HC doses by using gene sets (or modules) of co-expressed genes derived from the Modular Analysis Framework^20^ (Fig. 5a). Both HC doses down regulated mRNAs associated with inflammation and cell death modules at four hours (Fig. 5a), with the 50 mg dose exhibiting this change as early as one hour. HC down regulated expression of genes in the NF-κB signaling pathway including toll-like receptor (TLR) mRNAs at four hours (Fig. S4), with both doses exerting a similar magnitude of effect. In contrast, apoptotic signaling transcripts were up-regulated at 12 hours, and HC significantly up regulated cell cycle related mRNAs at four and eight hours, An NK/T cell activation module was markedly up-regulated as early as one hour after HC infusion (Figs 5a and 6). The profile of top genes associated with NF-κB signaling (Fig. S4) closely correlated with changes in monocyte and lymphocyte counts (Fig. 1). Upregulation of NF-κB and Toll-like receptor genes correlated with monocyte and lymphocyte counts at 12 hours, but not at other time points (Figs S5 and S6). We identified a signature of cell cycle control genes that exhibit a dose-responsive effect on expression, with a predominant pattern of down regulation after 50 mg HC and upregulation after 250 mg HC (Fig. S7).

We performed GSEA analysis of genes in the glucocorticoid receptor (GR) pathway available in the GeneGo database. We found that this gene set is enriched by down-regulated genes at 1 and 4 hours after HC administration (data not shown). To illustrate this enrichment we analyzed the differentially expressed genes through the use of QIAGEN’s Ingenuity® Pathway Analysis which describes significant changes in transcripts associated with GR signaling, and organizes them functionally with respect to their effects on inflammation. As shown in Fig. S8, HC exerted pleotropic effects on glucocorticoid signaling effectors, with a composite effect of down regulating inflammation. HC upregulated CD163, ADRB2, and IL1R2 transcripts, which favor an anti-inflammatory response, while down regulating NF-κB, c-Fos, and c-Jun signaling transcripts, as well as several of their downstream effectors that enhance inflammation (Fig. S8).

Using cell population specific gene sets defined by Nakaya *et al.*^21^, we detected a global decrease in monocyte and dendritic cell gene expression within the first eight hours after HC infusion, and activation of NK gene expression within this time frame (Fig. 5b). T cell genes were decreased between eight and twelve hours (Fig. 5b). We analyzed the correlation between coherent changes in cell populations as measured by flow cytometry and gene expression, with the top 50 up regulated and down regulated genes correlated with cell populations exhibiting the greatest changes from baseline. As shown in Fig. S9, HC enhanced expression of several NK/T cell transcripts in NK cells, including KLRC-1,2,3,4 and KIR3DL2.

### Effects of systemically administered HC on plasma cytokine levels

**Figure 7:**
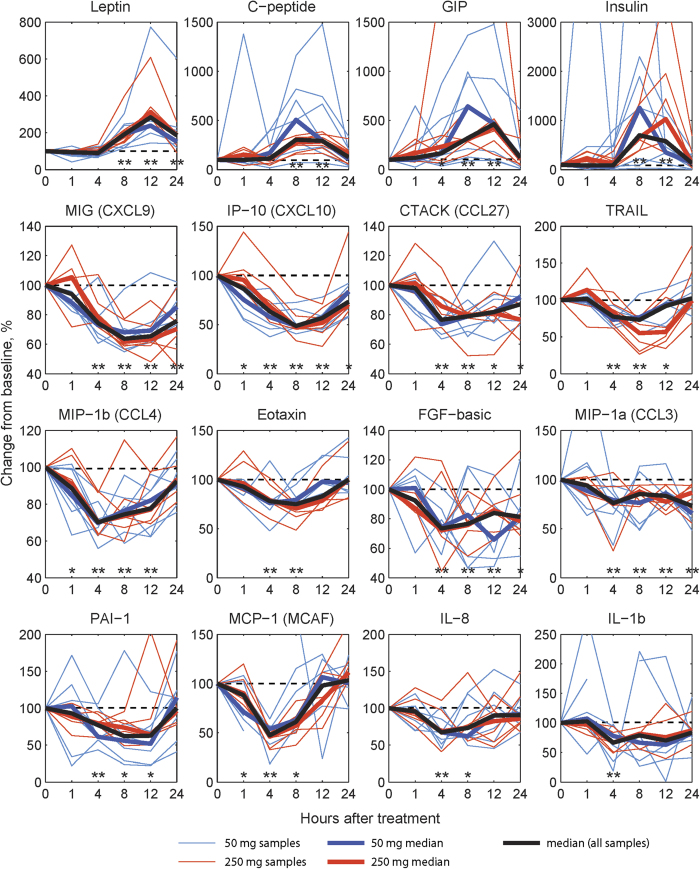
Cytokine levels as measured by Luminex assay after HC administration. Results are shown as the percentage changes from baseline concentration for each person. Thin lines indicate individual patients received HC dosage represented by color. Median changes for each dosage as well as for all the patients are shown as thick lines. Significance of changes from the baseline were estimated by nonparametric analyses with Wilcoxon matched pairs (signed-rank) test for all patients together. For each cytokine time points with *p* values lower than 0.05 are marked with*, and those with Benjamini-Hochberg FDR lower than 0.05 are marked with**.

We measured plasma cytokine levels using Luminex assay to determine the effects of HC on circulating human cytokine levels. To minimize intra-subjects and inter-plate variations, all concentrations were expressed as percentage of baseline, each subject serving as its own control. Among 67 analytes examined, only four cytokines, leptin, C-peptide, GIP, and insulin were significantly increased after HC administration, all peaking 8-12 hours after both HC doses (Fig. 7). All significant changes occurred within the first 24 hours after infusion.

Of cytokines measured, 12 showed significant decreases from baseline including IFN response proteins and inflammatory cytokines; PAI-1, MCP-1, IP-10, MIG, CTACK, MIP-1a, MIP-1b, TRAIL, eotaxin, IL-1b, IL-8, and FGF-basic. Most changes occurred at 4, 8, and 12 hours (Fig. 7). IP-10, MCP-1 and MIP-1b were decreased in the first hour.

## Discussion

Our objective was to evaluate the cellular and molecular effects of systemically administered corticosteroids in humans. We utilized HC because it is metabolized uniformly in humans, and moderate (250 mg) and low (50 mg) doses of hydrocortisone were selected because they are commonly prescribed.

Previous studies demonstrated that corticosteroids exert pleiotropic effects on cellular immunity, including margination of neutrophils, depletion of monocytes, dendritic cells, and CD4+CD8+ thymocytes by apoptosis, and mobilization of natural killer cells and regulatory T cells^4^,^5^,^7^. *In vitro* studies also showed that corticosteroids down modulate diverse inflammatory cytokines^4^,^5^,^6^,^7^,^15^, and they influence intracellular cytokine expression in T cells, resulting in an increased Th2/Th1 ratio^4^,^5^,^22^. Inhibition of cytokine production by glucocorticoids *in vitro* occurs through transcriptional repression, mRNA instability, as well as post-transcriptional mechanisms^4^,^5^. Many studies used concentrations of glucocorticoids that are higher than those achieved clinically.

Fauci *et al.* reported that 400 mg and 100 mg doses of HC cause a transient nadir in circulating peripheral blood lymphocytes at 4–6 hours and recovery to baseline after 24 hours^10^. Our data are consistent with these observations. We also observed differential effects of the two steroid doses on lymphocyte recovery at 24 hours, with total T cells, CD4+ T cells, and B cells rebounding above baseline after the 250 mg dose and total NK cells showing no increase above baseline at 24 hours. HC induced a rapid decline in circulating monocytes and mRNAs related to innate immune signaling as early as one hour after infusion. These effects preceded neutrophil demargination and lymphocyte depletion.

To further investigate the effects of HC on lymphocyte subsets at the four hour nadir, we utilized comprehensive lymphocyte immune phenotyping, a multiplexed 15-color flow cytometry assay which allowed us to examine 120 discrete lymphocyte and NK cell populations^18^,^19^. HC exerted markedly pleiotropic effects on lymphocyte subsets, with T cells showing the most pronounced changes. Total and CD4+ T cell frequencies decreased after both steroid doses, with no effect on CD4+CD8+ T cell frequencies in contrast to *in vitro* studies^4^. HC exerted differential effects on frequencies of CD4+ T cell subsets, with a decrease in naïve T helper subsets and an increase in effector and memory T helper cells.

Th17 cells exert pleiotropic effects on immunity through secretion of the pro-inflammatory cytokine IL-17, and they have been widely implicated as contributors to the pathogenesis of human autoimmune and inflammatory diseases, and graft versus host disease^23^,^24^,^25^,^26^. *In vitro* and animal studies demonstrate that Th17 cells increase after corticosteroid exposure^27^, and the mediate resistance to glucocorticoid therapy^28^,^29^. We observed that circulating Th17 cells were increased after systemic HC, consistent with *in vitro* and animal studies^27^,^28^,^29^.

Corticosteroids have been reported to increase Tregs in patients with inflammatory diseases and in *in vitro* studies, a finding that has been postulated to explain the immune modulatory effects of these agents^4^,^30^. However, we observed no changes in total Treg levels at 4 or 24 hours after HC, consistent with a recent report in human subjects treated for acute hearing loss^31^, and the call into question the role of Tregs in the immune suppressive effects of corticosteroids in humans. Similarly we did not observe an increase in Th2:Th1 polarization after HC administration, as prior *in vitro* and animal studies have demonstrated^4^,^27^, nor did we observe upregulation of the Th2 cytokine transcripts c-maf and IL4 as has been observed *in vitro*^16^. Serial or prolonged dosing of HC may induce a tolerogenic T helper cell phenotype, there is currently no evidence that this phenomenon observed *in vitro* occurs in healthy humans.

In contrast to the effects on T cells, HC exerted few quantitative changes in B cell subsets, with no change in B cell numbers four hours after HC administration. There was also relatively little effect of HC on B cell specific gene expression. Both total and immature NK cell frequencies were increased at four hours, while immature NKs decreased after HC infusion. Despite relatively few changes in frequency of NK subsets after HC exposure, we observed a striking effect on NK cell signaling genes in whole transcriptome analysis: NK cell activation genes KIR3DL2, KLRC3, KLRD1, and GPR56 were up-regulated as early as one hour after HC infusion, with a sustained up regulation of NK genes observed for eight hours. The finding that HC exerts activating effects on NK signaling transcripts has implications for the therapeutic use of corticosteroids when NK function is desired, as for adoptive cell therapy for malignancies^32^,^33^,^34^, and to preserve NK cell graft-versus-tumor effect after hematopoietic stem cell transplant^32^,^35^.

Glucocorticoids induce apoptosis in lymphocytes through a mechanism that is not fully understood, but is inhibited by Bcl-2 and Bcl-X and requires caspase-9^36^,^37^,^38^, suggesting at least partial mediation by the mitochondrial pathway. We observed an initial down regulation of transcripts associated with apoptosis and cell death signaling (modules 6.6, 6.13) correlating closely with absolute lymphocyte counts immediately after HC administration, followed by later up regulation, suggesting that HC exerts pro-apoptotic effects through modulation of gene expression.

*In vitro* and animal studies demonstrate that NF-κB exerts anti-apoptotic effects through signaling through downstream effectors^39^,^40^,^41^. One putative mechanism by which corticosteroids suppress immunity is down regulation of the NF-κB signaling^39^,^40^,^41^, an effect that enhances apoptosis. However, to our knowledge, no previous study has evaluated this mechanism in humans. We observed that NF-κB signaling was down-regulated as early as one hour in PBMCs after systemic HC administration, with peak inhibition occurring at 4–8 hours and both doses exerting a similar magnitude of inhibition. While the changes in NF-κB and TLR signaling pathways correlate to the changes in monocyte and lymphocyte populations, our analysis indicates that cellular composition is not the primary cause of these transcription changes. Our findings support published *in vitro* and animal studies, and suggest that inhibition of NF-κB signaling also contributes to the immune modulatory effects of HC in humans.

A limitation of our study is that we are only able to measure cells in the peripheral blood, so we are unable to determine the mechanism of the cell population changes that we observe. Previous studies using radiolabelled isotopes have demonstrated an effect of corticosteroids on neutrophil demargination from the bone marrow, and recirculation of lymphocytes to immune compartments^9^. We hypothesize that these mechanisms predominantly contribute to the changes in circulating cells present after HC infusion, based on the rapid time course of population changes we observed. Similarly, our sample size was not large enough to perform a robust deconvolution analysis such as csSAM to precisely determine which cell populations exhibit changes in gene expression.

Our study confirms in humans *in vitro* and animal studies which demonstrate that glucocorticoids down-regulate inflammatory cytokine levels^4^,^5^,^6^,^7^,^16^. Systemic HC infusion induced an inhibitory effect on transcription modules associated with inflammation at early time points, including several transcripts within the glucocorticoid-receptor signaling cascade. These data suggest that the anti-inflammatory effects of glucocorticoids occur through modulation of mRNA levels in humans. Our studies are also consistent with *in vitro* data^16^ demonstrating that glucocorticoids also down regulate transcripts associated with T cell chemotaxis (CXCL10), adhesion (CD36), and apoptosis (TNFSF10), oxidative function (CYBB), and genes upregulated by IFN-γ (WARS). IP-10 and MCP-1 exhibited the most pronounced responsiveness to HC administration. Circulating IL8 levels were decreased after HC infusion, during the time frame that neutrophil demargination occurred. IL8 is a pro-inflammatory cytokine that promotes neutrophil chemotaxis^42^. Our data suggest that chemotaxic mechanisms other than IL8 modulate neutrophil demargination in response to HC in humans. As anticipated, 50 mg HC increased leptin, GIP, and c-peptide levels, indicated that low dose glucocorticoids exert this effect in humans.

In summary, we have performed a comprehensive interrogation of the human immunome in healthy volunteers exposed to doses of HC prescribed in clinical practice. We report that HC exerts differential effects on circulating populations of lymphocyte and NK cells, with the most profound effects on T cell subsets, and significant changes in genes whose products play key roles in lymphocyte and NK signaling, as well as cell death and apoptosis signaling. These observations provide a new level of insight into the immune effects of the widely prescribed class of therapeutics.

## Materials and Methods

### Clinical protocol

Healthy volunteers older than 18 years were enrolled on NIH protocols 11-H-0092 and 09-H-0201, both approved and monitored by NHLBI, NIH institutional review boards in accordance with the Declaration of Helsinki and registered under clinicaltrials.gov (NCT01281995 registered January 21, 2011; and NCT00968084 registered August 27, 2009). Healthy volunteers were screened to exclude occult autoimmune and inflammatory conditions. Pregnant subjects and those who had received vaccines or taken immune modifying medications within six months were excluded. Informed consent was obtained from all subjects.

We enrolled twenty healthy volunteers, median age 33 (range 22–63), 70% Caucasian, 10% African American, 15% Asian, and 5% Hispanic, and evenly divided between sexes. Subjects had baseline phlebotomy (150 mL), and were then administered a single dose of 50 mg or 250 mg IV hydrocortisone. Blood was collected at baseline immediately prior to receiving hydrocortisone (or 0), one, four, eight, 12, and 24 hours after infusion, as well as on days seven (+/− two days), and 28 (+/− two days) after HC infusion.

### Clinical flow cytometry

Blood samples were stained and analyzed using a four color automatic flow cytometer and software from Beckman Coulter. Briefly, whole blood was stained with directly conjugated monoclonal antibodies for CD3, CD4, CD8, CD16, CD56, CD19, and CD45 followed by a red cell lysis. Lymphocytes were gated using CD45 and side scatter. Percentages and absolute counts of CD3+ T cells, CD19+ B cells, and CD16/CD56+ NK cells were determined.

### Flow cytometry for comprehensive immunophenotyping

PBMCs were prepared by Ficoll separation and cryopreserved at −120 degrees Celsius according to CHI protocols (http://www.nhlbi.nih.gov/resources/chi/documents/SOP-Isolation.pdf). After thawing, cells were washed once and re-suspended in PBS. Viability staining was performed for 15 min in presence of LIVE/DEAD Aqua fixable viability dye (Life Sciences, Carlsbad, CA) followed by a wash in FACS staining buffer (PBS supplemented with 1% normal mouse serum, 1% goat serum and 0.02% sodium azide) (Gemini Bioproducts, West Sacramento, CA). Cells were stained according to our protocols^18^,^19^ for five tubes of the CLIP panel (T_reg_, Th_17_, Th_1_/Th_2_, B_naive/memory_ and NK cells) (Table S1). Three time points per patient were acquired on the same day using one common mixture of antibodies. Acquisition was performed using a Becton Dickinson LSRFortessa (BD, San Jose, CA) equipped with five lasers (355 nm, 407 nm, 488 nm, 532 nm, and 633 nm wavelengths) with 22 PMT detectors, optimized as described by Perfetto *et al.*^43^. Data were acquired using DIVA 6.1.2 software (BD) and we ensured a minimum of 50, 000 CD4 T cells was recorded to be able to accurately assess minor cell populations. Compensation was performed with unstained cells and BD compensation beads particle sets (using only the positive beads). While compensation was used during acquisition of the specimen to ensure recording of enough events for the populations of interest, a final compensation matrix was calculated using FlowJo version 9.6.2 (Treestar Inc., San Carlos, CA) during post-acquisition analysis. For the analysis, debris and doublets were excluded using light scatter measurements and major cell populations were identified based on their forward and side scatter properties as described^18^. Subsequently, viability stain and CD45 were used to ensure that only viable lymphocytes were included for analysis. Each cell population was represented as percentages of the parent population.

### Statistics

Frequencies of the manually gated cell populations were first transformed by log_10_ (all zero values were set to 0.01 before transformation). Samples and cell populations were assessed for data quality independently for each time point. Median of viable cell frequencies across all five tubes for all samples was above 80%. Cell populations having fewer than 20 cells in 80% of the samples were excluded from the subsequent analysis. Coherent changes in population abundance were statistically estimated using standard pairwise t-test. P-values were adjusted for multiple-testing using Benjamini and Hochberg’s method to estimate the false discovery rate (FDR)^44^.

### Whole transcriptome microarrays

Total RNA was extracted from PBMCs using a miRNeasy kit (Qiagen, Valencia, CA). Universal RNA quality and quantity was estimated using Nanodrop (Thermo Scientific, Wilmington, DE) and Agilent 2100 Bioanalyzer (Agilent Technologies, Palo Alto, CA). RNA was amplified from 300 ng of total RNA (Ambion WT Expression Kit). cDNA was reverse transcribed with biotinylation and hybridized to the GeneChip Human Gene 1.0 ST Arrays (Affymetrix WT Terminal Labeling Kit) after fragmentation. The arrays were washed and stained on a GeneChip Fluidics Station 450 (Affymetrix, Santa Clara, CA); scanning was carried out with the GeneChip Scanner 3000 and image analysis with the Affymetrix GeneChip Command Console Scan Control.Microarray data used in this manuscript are available in the NCBI Gene Expression Omnibus—GEO (GSE67255; http://www.ncbi.nlm.nih.gov/geo/query/acc.cgi?acc=GSE67255).

### Statistical methods for microarray data

Affymetrix CEL files were processed with Affymetrix Power Tools for probeset summarization, normalization and log2-transformation (RMA with sketch quantile normalization). Quality assessment was conducted using R/Bioconductor package “arrayQualityMetrics”^45^ and no outlying arrays were found. Array hybridization date was found to be significantly correlated with most probe sets and its effect was removed using linear regression with R “limma” package^46^.

Probesets without annotated gene symbol (using the latest NetAffx annotation) as well as probesets with very low variation across samples (with inter-quantile range across all samples less than 0.15) were filtered out. In addition, probesets associated with multiple gene symbols were also removed. In the case where a gene is associated with multiple probesets, we selected the probeset having the highest correlation with the first principal component computed from all probesets across all the samples (in-house developed R function). After all filtering steps 15166 genes were left.

To determine genes whose expression changed significantly from baseline the R/Bioconductor package “limma”^46^ was used. Subject IDs were included into the model to perform paired analysis – i.e., to ensure that individual baselines were used. P-values were adjusted for multiple-testing using Benjamini and Hochberg’s false discovery rate (FDR) method^44^. The cutoffs used to identify differentially expressed genes were: <5% FDR-adjusted p-value and >0.5 absolute average log2-fold-change. Correlation between changes in gene expression from baseline and changes in cell counts were performed using non-parametric Spearman correlation.

### Gene set enrichment analysis

To assess gene function or cell compositional enrichments from blood transcriptomic changes, we utilized blood transcriptomic gene modules developed by Chaussabel and colleagues^20^,^47^. These modules were determined based on genes co-expressed across expression profiles from one or more blood transcriptomic data sets. The annotation and content of these modules can be found at http://www.biir.net/public_wikis/module_annotation/V2_Trial_8_Modules. To determine if our coherently changing genes are enriched for a predefined set of genes associated with a particular transcriptional module, we applied gene set enrichment analysis^48^,^49^ that ranks all genes based on a statistic (e.g., -log(p-value) from differential expression) and then evaluates whether genes from a given geneset tend to be located near the top of the ranked list than that expected by chance. We utilized a variation of this strategy implemented as the *geneSetTest* function in the limma R/Bioconductor package^46^. The ranking of genes was first computed according to: (1−*p*) * 
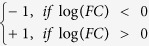
, to uncover gene sets enriched for up-regulated genes, then the ranked list was reversed to assess for enrichment involving down-regulated genes. Here *p* represents p-values from differential gene-expression analysis of changes from time-point X relative to baseline (day 0 – prior to steroid administration). We used PBMC cell specific gene sets from Nakaya *et al.* to evaluate gene enrichment associated with monocytes, T, B, NK, and dendritic cells^21^.

### Luminex assay

Plasma was collected and cryopreserved at −120 degrees Celsius according to CHI protocols (http://www.nhlbi.nih.gov/resources/chi/documents/SOP-Isolation.pdf). Luminex experiments were performed according to previously published methods^19^ using five different kits from Bio-Rad (Hercules, CA, USA): 27-plex cytokine group I, 21-plex cytokine group II, 10-plex diabetes, four-plex and five-plex acute phases according to Bio-Rad procedures. Hemolyzed plasma samples were excluded. Median fluorescence intensities were collected on a Luminex-100 instrument (Luminex, Bio-Rad), using Bio-Plex Manager software version 6. Standard curves for each cytokine were generated using lyophilized standards. Cytokine concentrations were determined from the appropriate standard curve using five point 5-parameter logistic regression to transform mean fluorescence intensities into concentrations. Samples were run in duplicate and the averages were used as the measured observed concentration. Statistical analysis of changes in cytokine level was performed for all subjects together by non-parametric analysis with Wilcoxon matched pairs (signed-rank) test. The resulted p-values were corrected for multiple testing with Benjamini-Hochberg FDR.

### Correlation analysis between gene expression and cell populations

We filtered significantly changed genes (FDR-corrected p-values < 0.05) and selected top 50 up and downregulated genes. Then we calculated Pearson correlation between log-fold-change in gene expression and cell populations for cells with the same change direction. We selected cell populations correlated with at least 5 genes with correlation coefficient greater than 0.3.

## Additional Information

**How to cite this article**: Olnes, M. J. *et al.* Effects of Systemically Administered Hydrocortisone on the Human Immunome. *Sci. Rep.* **6**, 23002; doi: 10.1038/srep23002 (2016).

## Supplementary Material

Supplementary Information
