# Corrigendum: Effects of Systemically Administered Hydrocortisone on the Human Immunome

**DOI:** 10.1038/srep25215

**Published:** 2016-05-04

**Authors:** Matthew J. Olnes, Yuri Kotliarov, Angélique Biancotto, Foo Cheung, Jinguo Chen, Rongye Shi, Huizhi Zhou, Ena Wang, John S. Tsang, Robert Nussenblatt, The CHI Consortium, Howard B. Dickler, Christopher S. Hourigan, Francesco M. Marincola, J. Phillip McCoy, Shira Perl, Paula Schum, Pamela L. Schwartzberg, Giorgio Trinchieri, Janet Valdez, Neal S. Young

Scientific Reports 6: Article number: 2300210.1038/srep23002; published online: 03142016; updated: 05042016

This Article contains errors in the Author Contributions Statement,

“J.S.T. headed and interpreted the data analysis, revised the manuscript.”

should read:

“J.S.T helped design data analysis strategies and analysis result interpretation, and contributed to manuscript revision.”

In addition, the present address for Francesco M. Marincola was omitted. The correct address is listed below:

Sidra Medical and Research Center, Research Branch, Doha, Qatar.

