# Supplementary material for: Effects of Systemically Administered Hydrocortisone on the Human Immunome
Source: Sci Rep. 2016 Mar 14;6:23002. doi: 10.1038/srep23002 (PMC4789739; doi:10.1038/srep23002)
Supplement: Supplementary Information [file srep23002-s1.pdf]

Effects of Systemically Administered Hydrocortisone on the Human Immunome

Matthew J Olnes[1,2,3*§], Yuri Kotliarov[1*], Angelique Biancotto[1], Foo Cheung[1], Jinguo Chen[1], Rongye Shi[1], Huizhi Zhou[1], Ena Wang[1,4], John S Tsang[1,5], and Robert Nussenblatt[1,6], and the CHI Consortium

The CHI Consortium:
Howard B Dickler[1], Christopher S Hourigan C[1,7], Francesco M Marincola[1,4], J Phillip McCoy[1], Shira Perl[1], Paula Schum[1], Pamela L Schwartzberg[1,8], Giorgio Trinchieri[1,9], Janet Valdez[1,2], Neal S Young[1,2]

1. Trans-NIH Center for Human Immunology, Autoimmunity, and Inflammation (CHI), National Institutes of Health (NIH), Bethesda, MD, USA 20892
2. Hematology Branch, National Heart, Lung, and Blood Institute (NHLBI), Bethesda, MD, USA, 20892
3. Presently at Hematology Department, Alaska Native Tribal Health Consortium, Anchorage, AK, USA, 99508
4. Presently at Sidra Medical and Research Centre, Doha, Qatar
5. Systems Genomics and Bioinformatics Unit, Laboratory of Systems Biology, National Institute of Allergy and Infectious Diseases, NIH, Bethesda, MD, USA 20892
6. Laboratory of Immunology, National Eye Institute, NIH, Bethesda, MD, USA 20892
7. Myeloid Malignancies Section, NHLBI, NIH, Bethesda, MD, USA, 20892
8. Medical Genetics Branch, National Human Genome Research Institute, NIH, Bethesda, MD, USA, 20892
9. Program in Cancer and Inflammation, National Cancer Institute, NIH, Bethesda, MD, USA, 20892

[*] These authors contributed equally to this work

[§] Correspondence: Matthew J. Olnes, MD, PhD, FACP; Hematology Department; Alaska Native Tribal Health Consortium; Anchorage, USA, 99508. Phone (907) 729-1180, Fax (907) 729-1189, olnesmj@nhlbi.nih.gov

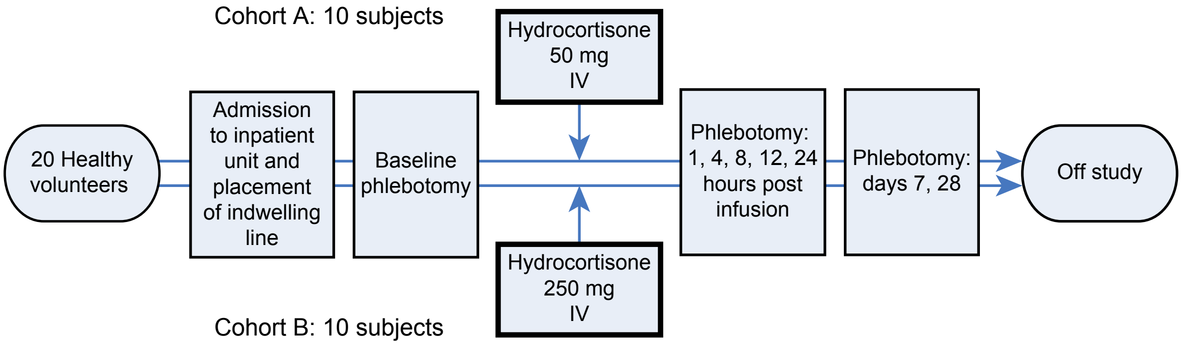

Figure S1. Clinical trial scheme.

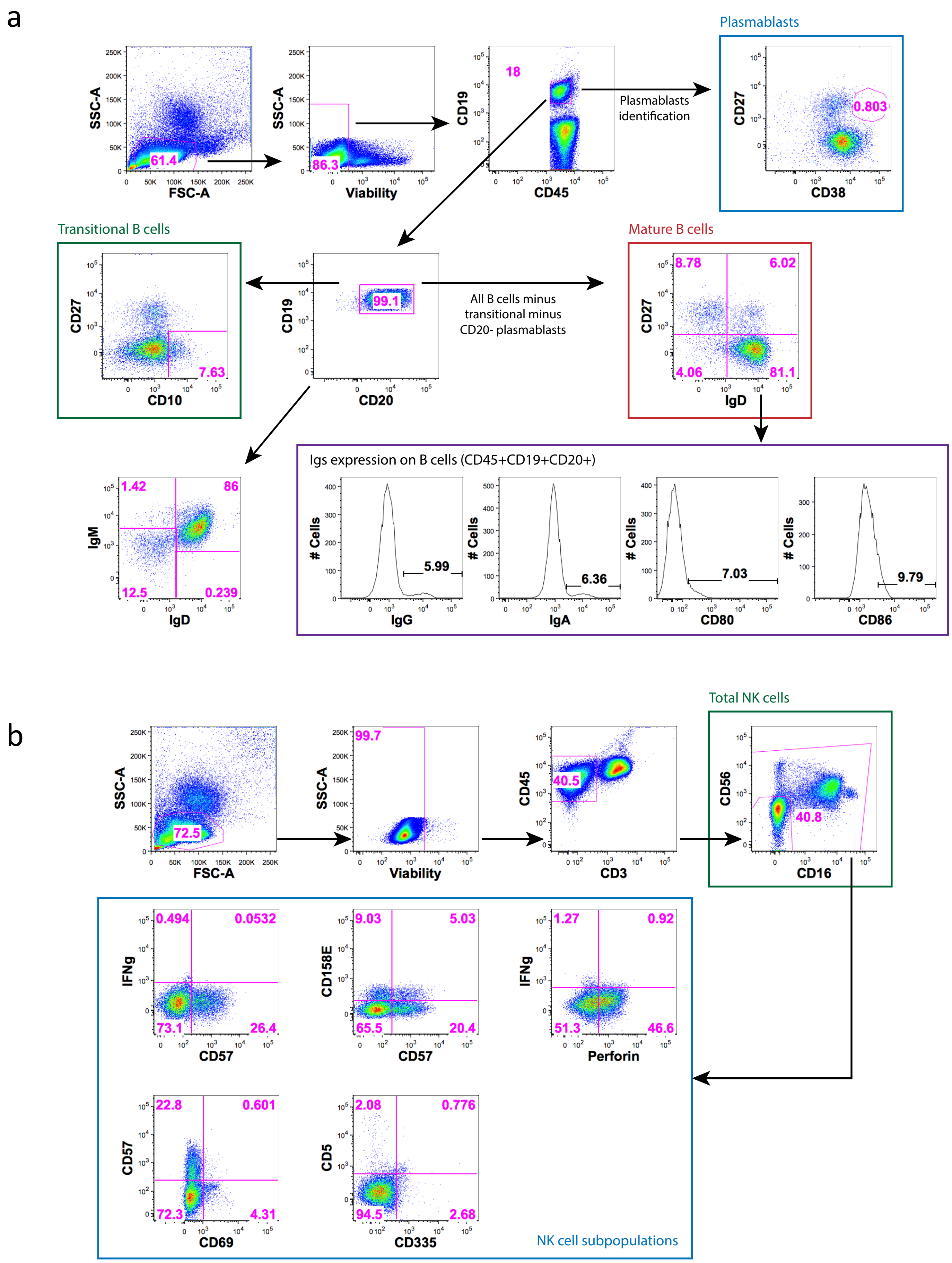

Figure S2. Staining patterns and gating strategies. (**a**) B cells lineage, (**b**) Natural Killer cells lineage.

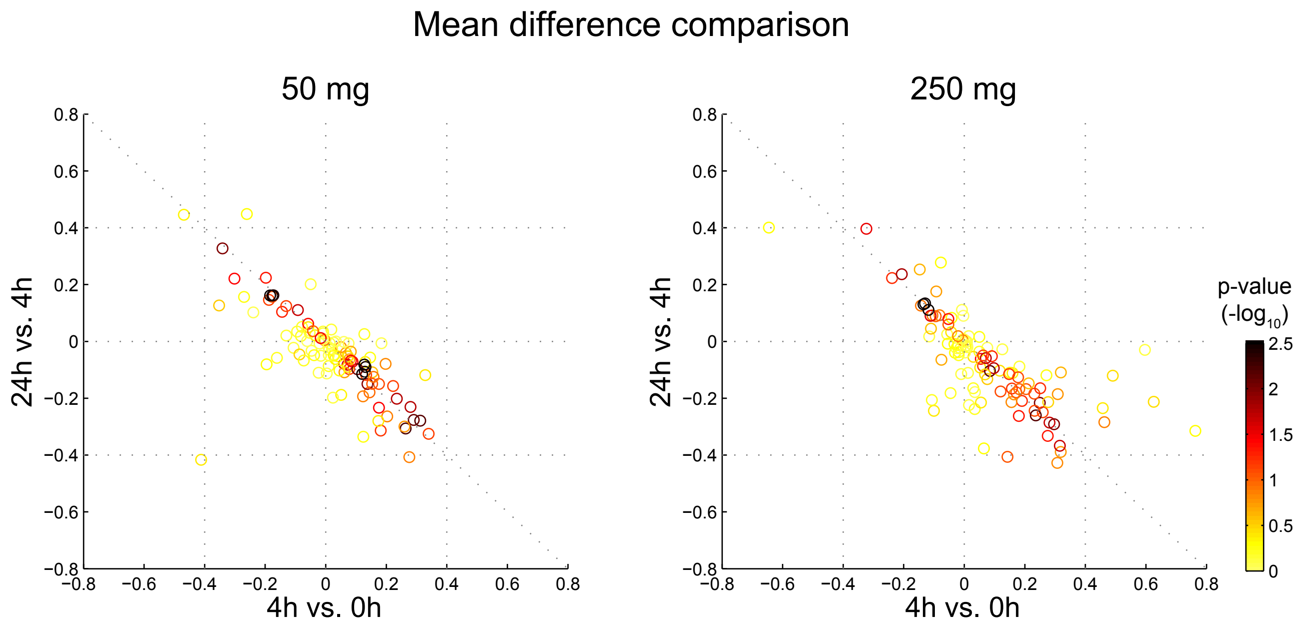

Figure S3. Virtually all characterized cell populations that exerted frequency changes at four hours returned to baseline values at 24 hours. Each dot represents cell population in the space of mean log-fold-change between 4 hours and 0 hour *vs*. mean log-fold-change between 24 hours and 4 hours. The dots are colored by logged t-test p-value for 4 hours change with black color corresponding to highest significance. The dots are well fitted on the negative identity line.

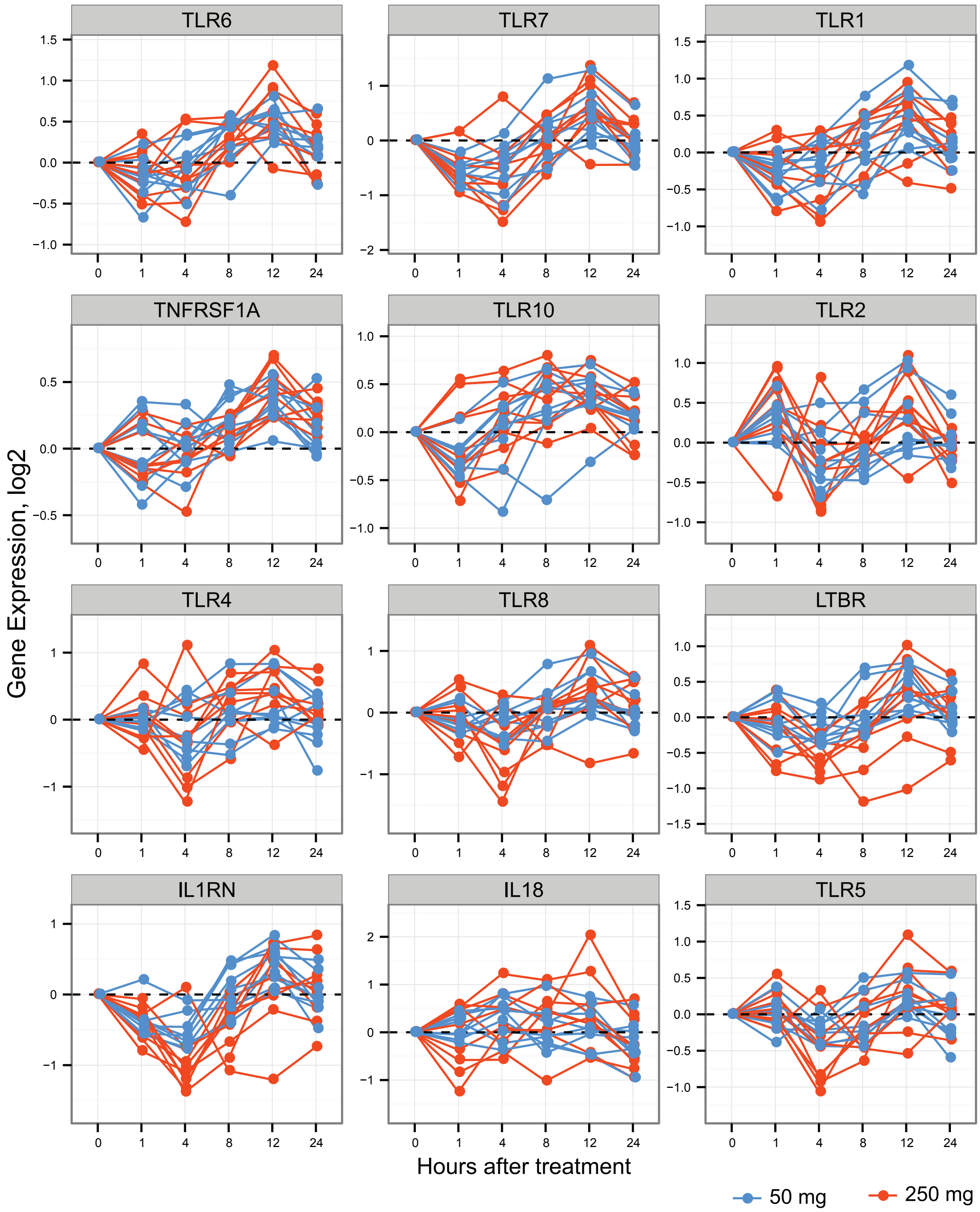

Figure S4. NF-κB gene signature changes after HC administration. Gene expression profiles of top genes from the NF-κB Signaling pathway (QIAGEN's Ingenuity® Pathway Analysis) with largest change from baseline to 12 hours. Each line represents an individual patient with blue and red color indicating patients receiving 50 or 250 mg of HC respectively.

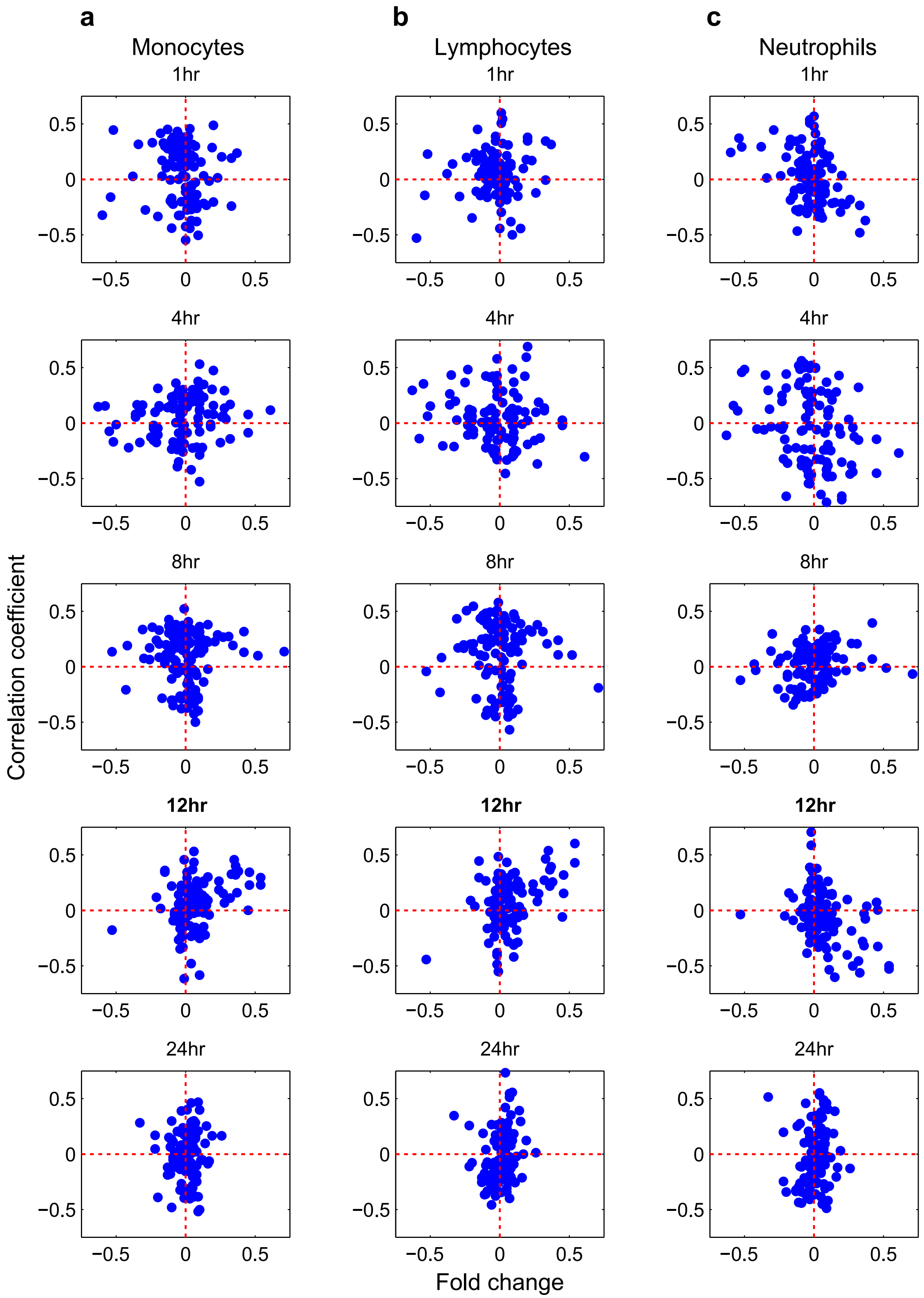

Figure S5. NF-κB related gene changes at 12 hours can be partially explained by increased amount of monocytes (a). Each scatter plot is correlation coefficient (Spearman) between changes in cell count and changes in gene expression from baseline (*y* axis) against changes in gene expression from baseline (*x* axis) at the indicated time point. Each point represents a gene from the NF-κB Signaling pathway (QIAGEN's Ingenuity® Pathway Analysis).

Similar pattern observed for **lymphocytes (b)** and reversed for **neutrophils (c)** due to how changes of these cells correlate with monocytes.

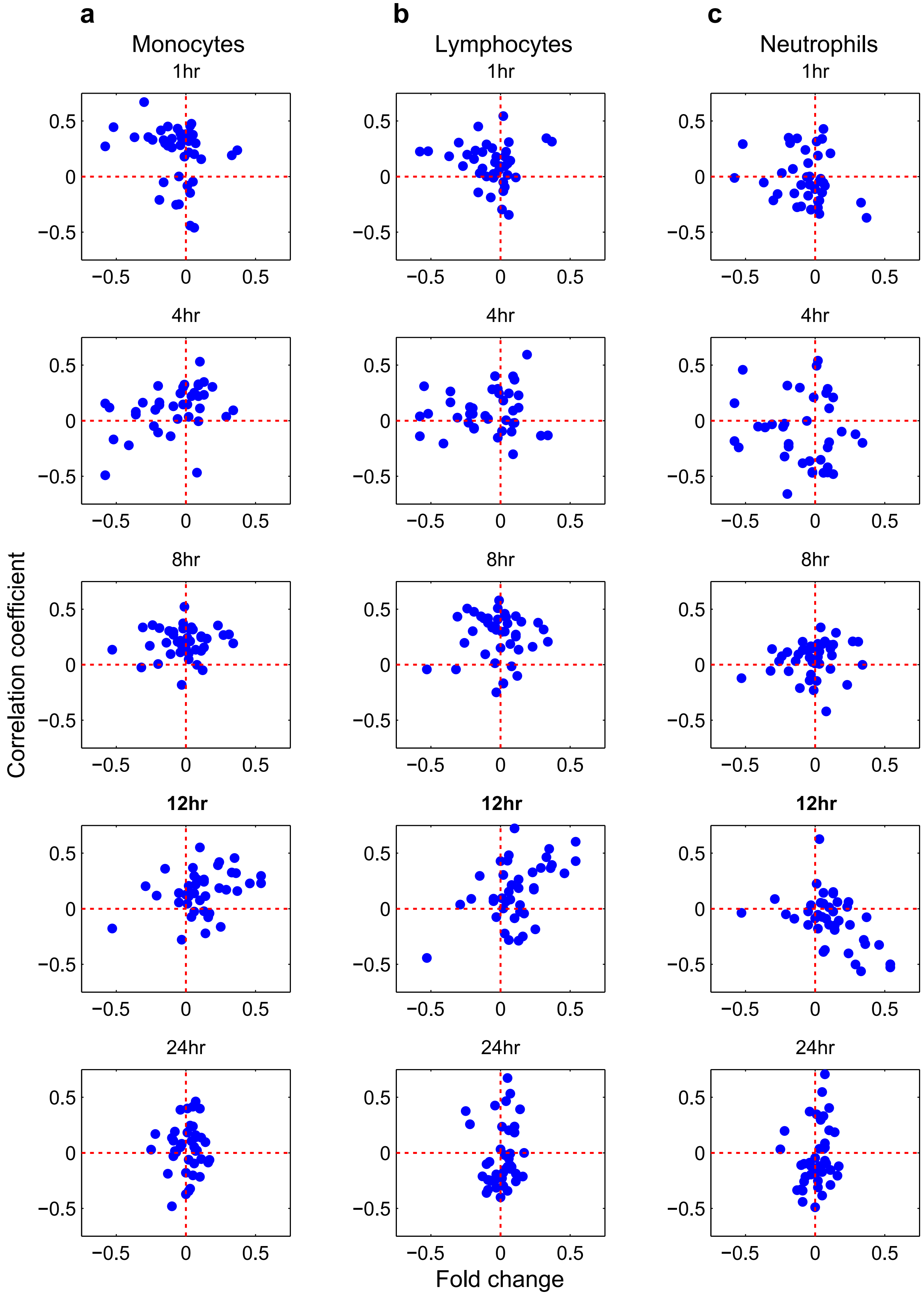

Figure S6. Toll-like Receptor Signaling gene changes at 12 hours can be partially explained by increased amount of monocytes (a). Each scatter plot is correlation coefficient (Spearman) between changes in cell count and changes in gene expression from baseline (*y* axis) against changes in gene expression from baseline (*x* axis) at the indicated time point. Each point represents a gene from the Toll-like Receptor Signaling pathway (QIAGEN's Ingenuity® Pathway Analysis).

Similar pattern is observed for **lymphocytes (b)** and reversed for **neutrophils (c)** due to how changes of these cells correlate with monocytes.

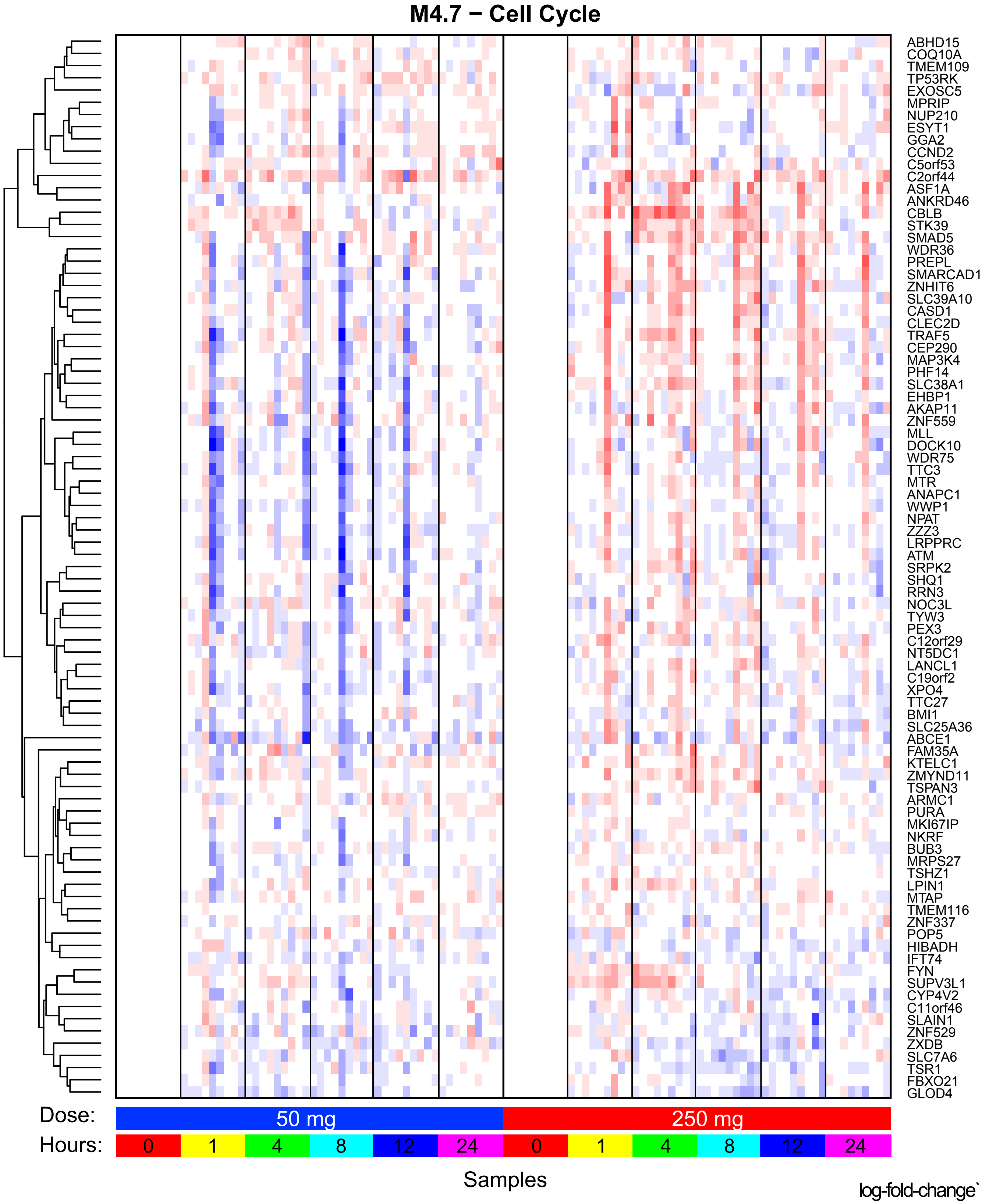

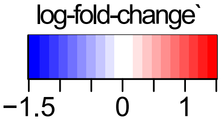

Figure S7. Coherent changes from baseline in expression of 97 genes in M4.7 (cell cycle) transcription module. This heat map shows that there to be a differential effect between low and moderate dose HC on expression of this gene set. We haven't observed this behavior in other cell cycle associated modules - M3.3, M6.11 and M6.16 (see Figure 5a). Each column represents a blood sample acquired at particular time point. The time points and HC dosages are annotated by colors.

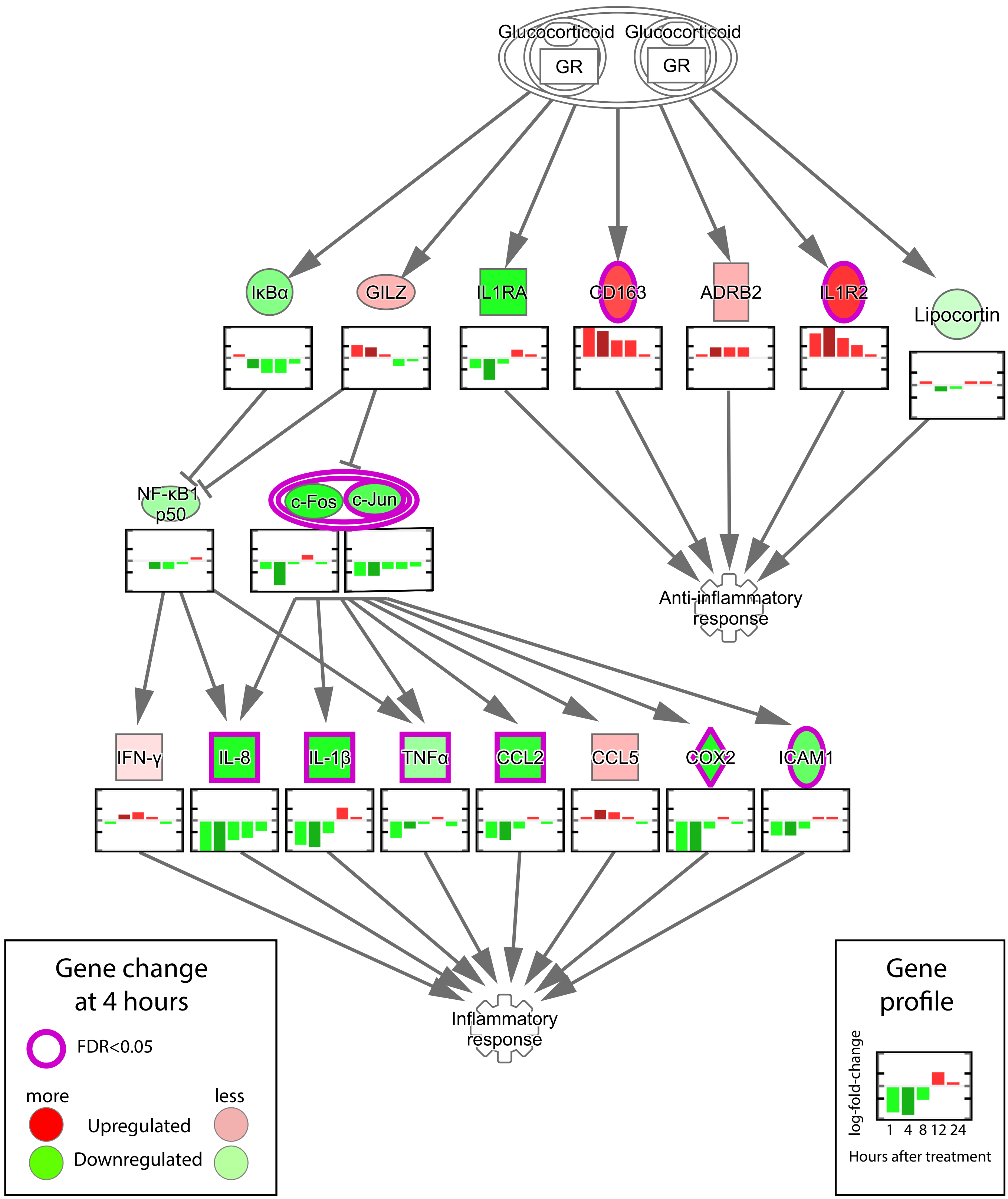

Figure S8. Glucocorticoid Receptor Signaling pathway genes with significant coherent changes at 4 hours. The network was generated through the use of QIAGEN's Ingenuity® Pathway Analysis. Gene colors represent their log-fold-change from the baseline at 4 hours. The barplots show fold-changes of each gene at all time points after HC administration. Node shapes pictured according to standard Ingenuity® legends (http://ingenuity.force.com/ipa/articles/Feature_Description/Legend).

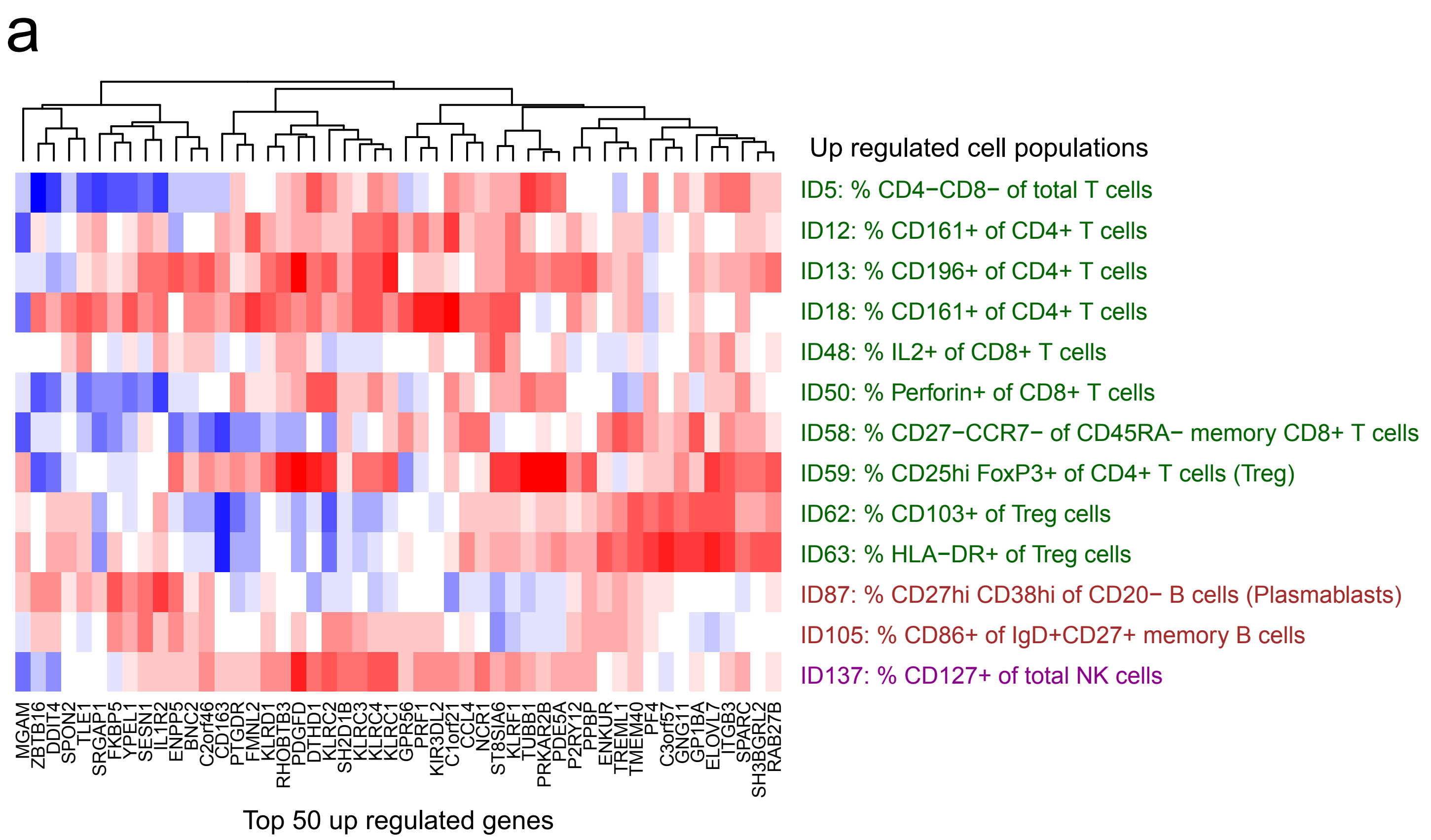

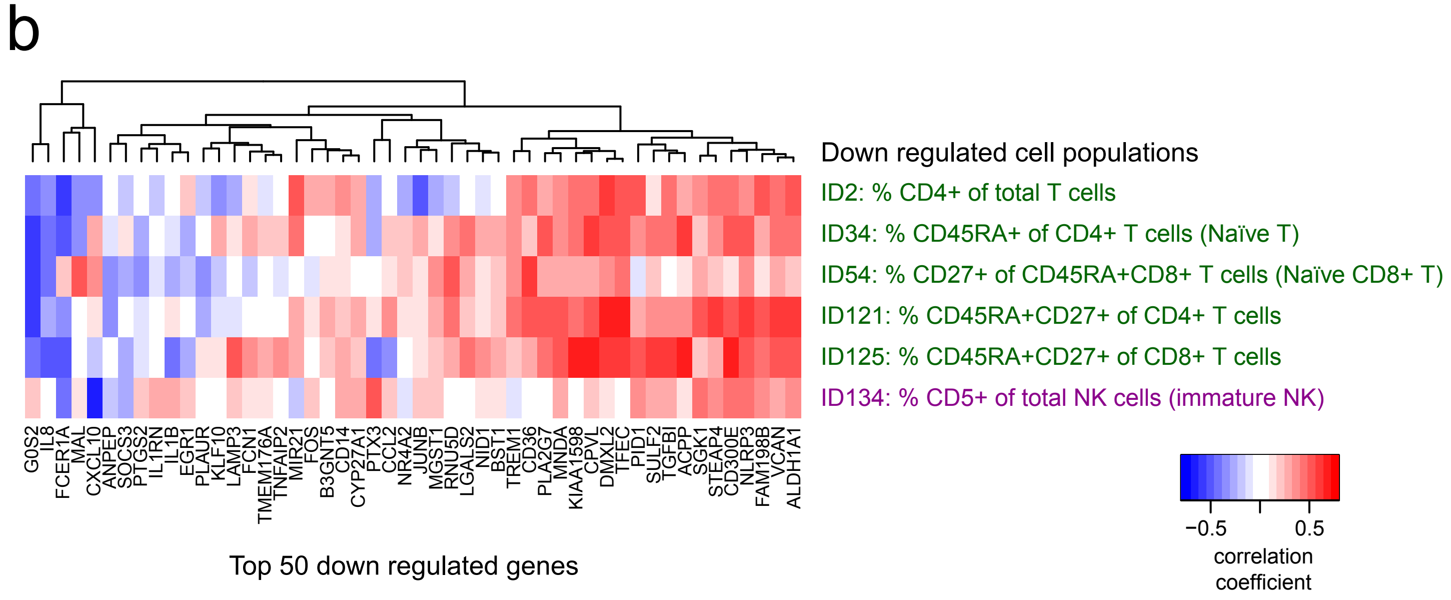

Figure S9. Correlations between coherent changes in gene expression and cell populations. Heat maps represent correlation coefficients for top 50 up- (**a**) and down-(**b**) regulated genes (FDR-corrected p-values < 0.05) and cell populations changed in the same direction. Cell populations are selected to be correlated with at least 5 genes with correlation coefficient greater than 0.3. The labels for T, B and NK cell populations are designated by green, brown and purple colors correspondingly.

Excitation (nm)	Fluorochrome name	PMT name	T lineage T_1	T_2	T_3	B lineage B_2	NK lineage NK_2
407	V450 Pac blue	**V450**	**CD4** *RPA-T4*	**CD4** *RPA-T4*	**CD4** *RPA-T4*	CD80 *L307.4*	**CD56** *B159*
	Aquablue	**V545**	**Viability**	**Viability**	**Viability**	**Viability**	**Viability**
	Qdot 605	**V605**	**CD8** *3B5*	**CD8** *3B5*	**CD8** *3B5*	CD27 *CLB-27/1*	**CD8** *3B5*
	Qdot 655	**V655**	**CD27** *CLB-27/1*	**CD27** *CLB-27/1*	**CD27** *CLB-27/1*	**CD19** *SJ25-C1*	**NKp46** *900*
	Qdot 800	**V800**	**CD45** *HI30*	**CD45** *HI30*	**CD45** *HI30*	**CD45** *HI30*	**CD45** *HI30*
488	FITC	**B515**	CD39 *A1*	IL-23R *218213*	CD69 *FN50*	IgA *Polyclonal Goat*	CD57 *TB01*
	PcPcy5.5	**B710**	CD38 *HIT2*	CD196 *11A9*	IL-4 *MP4-25D2*	CD86 *IT2.2*	IFNg *4S.B3*
532	PE	**G560**	Foxp3 ***PCH101***	IL-22 *142928*	perforin *δG9*	CD21 *BL13*	Perforin *δG9*
	PE-TR	**G610**	**CD45-RA** *2H4LDH11LDB9*	**CD45-RO** *UCHL1*	**CD45-RO** *UCHL1*	**CD38** *HIT2*	**CD25** *B1.49.9*
	PEcy5	**G660**	CD103 *LF61*	CD161 *DX12*	CD40L *TRAP1*	IgG *G18-145*	CD16 *3G8*
	PEcy5.5 /PE-A700	**G710**	HLA-DR *Tu36*	TNFa *MP9-20A4*	TNFa *MP9-20A4*	**CD20** *HI47*	**CD69** *CH/4*
	PEcy7	**G780**	CD25 *M-A251*	IL-17A *BL168*	IFNg *4S.B3*	CD23 *EBVCS-5*	**CD5** ***L17F12***
633	APC	**R660**	CD127 *hIL-7R-M21*	IL-21 *3A3-N2.1*	IL-2 *MQ1-17H12*	IgD *IgD26*	CD127 *hIL-7R-M21*
	Alexa700/APC Cy5.5	**R710**	**CD197** *150503*	**CD197** *150503*	**CD197** *150503*	IgM *CH2*	CD158e1 *DX9*
	APC cy7	**R780**	**CD3** *Sk7*	**CD3** *Sk7*	**CD3** *Sk7*	CD10 *HI10a*	CD3 *Sk7*

**Lineage mix**	
**CD3**	**SK7**
**CD56**	**NCAM16.2**
**CD20**	**L27**
**CD19**	**SJ25C1**
**CD16**	**3G8**

Table S1. The design of the flow cytometry panels used in this study to assess cell population frequencies.

ID	Tube	Parent Population	Markers	Category	Populations Name
ID1	T1	(as of viable CD45+)	CD45+CD3+	T cells	% CD3+ of viable CD45+ cells (Total T cells)
ID2	T1	(as of viable CD45+CD3+) quadrant	CD45+CD3+CD4+ CD8-	T cells	% CD4+ of total T cells
ID3	T1	(as of viable CD45+CD3+)	CD45+CD3+CD4+ CD8+	T cells	% CD4+CD8+ of total T cells
ID4	T1	(as of viable CD45+CD3+) quadrant	CD45+CD3+CD4- CD8+	T cells	% CD8+ of total T cells
ID5	T1	(as of viable CD45+CD3+)	CD45+CD3+CD4- CD8-	T cells	% CD4-CD8- of total T cells
ID6	T1, T2, T3	(as of viable CD45+CD3+) gated region	CD45+CD3+CD4+CD8-	T helper cells	% CD4+ of total T cells
ID7	T3	(as of viable CD45+CD3+ CD4+CD8-)	CD45+CD3+CD4+CD8-CD69+	T helper cells	% CD69+ of CD4+ T cells
ID8	T1	(as of viable CD45+CD3+ CD4+CD8-)	CD45+CD3+CD4+CD8-CD25+	T helper cells	% CD25+ of CD4+ T cells
ID9	T1	(as of viable CD45+CD3+ CD4+CD8-)	CD45+CD3+CD4+CD8-CD38+	T helper cells	% CD38+ of CD4+ T cells
ID10	T1	(as of viable CD45+CD3+ CD4+CD8-)	CD45+CD3+CD4+CD8-HLA-DR+	T helper cells	% HLA-DR+ of CD4+ T cells
ID11	T3	(as of viable CD45+CD3+ CD4+CD8-)	CD45+CD3+CD4+CD8-CD40L+	T helper cells	% CD40+ of CD4+ T cells
ID12	T2	(as of viable CD45+CD3+ CD4+CD8-)	CD45+CD3+CD4+CD8-CD161+	T helper cells	% CD161+ of CD4+ T cells
ID13	T2	(as of viable CD45+CD3+ CD4+CD8-)	CD45+CD3+CD4+CD8-CD196+	T helper cells	% CD196+ of CD4+ T cells
ID14	T2	(as of viable CD45+CD3+ CD4+CD8-)	CD45+CD3+CD4+CD8-IL17a+	T helper cells	% IL17+ of CD4+ T cells
ID16	T2	(as of viable CD45+CD3+ CD4+CD8-)	CD45+CD3+CD4+CD8-IL22+	T helper cells	% IL22+ of CD4+ T cells
ID17	T2	(as of viable CD45+CD3+ CD4+CD8-)	CD45+CD3+CD4+CD8-IL23r+	T helper cells	% IL23R+ of CD4+ T cells
ID18	T2	(as of viable CD45+CD3+ CD4+CD8-) histogram	CD45+CD3+CD4+CD8-CD161+	T helper cells	% CD161+ of CD4+ T cells
ID22	T2	(as of viable CD45+CD3+ CD4+CD8-) histogram	CD45+CD3+CD4+CD8-CD161-	T helper cells	% CD161- of CD4+ T cells
ID26	T3	(as of viable CD45+CD3+ CD4+CD8-)	CD45+CD3+CD4+CD8-IL2+	T helper cells	% IL2+ of CD4+ T cells
ID27	T3	(as of viable CD45+CD3+ CD4+CD8-)	CD45+CD3+CD4+CD8-IFNg+	T helper cells	% IFNg+ of CD4+ T cells
ID28	T3	(as of viable CD45+CD3+ CD4+CD8-)	CD45+CD3+CD4+CD8-IL4+	T helper cells	% IL4+ of CD4+ T cells
ID29	T3	(as of viable CD45+CD3+ CD4+CD8-)	CD45+CD3+CD4+CD8-TNFa+	T helper cells	% TNFa+ of CD4+ T cells
ID30	T1	(as of viable CD45+CD3+ CD4+CD8-)	CD45+CD3+CD4+CD8-CD39+	T helper cells	% CD39+ of CD4+ T cells
ID31	T1	(as of viable CD45+CD3+ CD4+CD8-)	CD45+CD3+CD4+CD8-CD103+	T helper cells	% CD103+ of CD4+ T cells
ID32	T1	(as of viable CD45+CD3+ CD4+CD8-)	CD45+CD3+CD4+CD8-CD127+	T helper cells	% CD127(IL7R)+ of CD4+ T cells
ID33	T1	(as of viable CD45+CD3+ CD4+CD8-)	CD45+CD3+CD4+CD8-CD27+	T helper cells	% CD27+ of CD4+ T cells
ID34	T1	(as of viable CD45+CD3+ CD4+CD8-)	CD45+CD3+CD4+CD8-CD45RA+	T helper cells	% CD45RA+ of CD4+ T cells (Naïve T cells)
ID35	T1	(as of viable CD45+CD3+ CD4+CD8-)	CD45+CD3+CD4+CD8-CD45RA-	T helper cells	% CD45RA- of CD4+ T cells (Total memory CD4+ T cells)
ID36	T1	(as of viable CD45+CD3+ CD4+CD8- CD45RA-)	CD45+CD3+CD4+CD8-CD45RA-CD27+CCR7-	T helper cells	% CD27+CCR7- of memory CD4+ T cells (Effector
ID37	T1	(as of viable CD45+CD3+ CD4+CD8- CD45RA-)	CD45+CD3+CD4+CD8-CD45RA-CD27+CCR7+	T helper cells	% CD27+CCR7+ of memory CD4+ T cells (Central
ID38	T1, T2, T3	(as of viable CD45+CD3+) gated region	CD45+CD3+CD8+CD4-	T cytotoxic cells	% CD8+ of total T cells
ID39	T3	(as of viable CD45+CD3+CD8+CD4-)	CD45+CD3+CD8+CD4-CD69+	T cytotoxic cells	% CD69+ of CD8+ T cells
ID40	T1	(as of viable CD45+CD3+CD8+CD4-)	CD45+CD3+CD8+CD4-CD25+	T cytotoxic cells	% CD25+ of CD8+ T cells
ID41	T1	(as of viable CD45+CD3+CD8+CD4-)	CD45+CD3+CD8+CD4-CD38+	T cytotoxic cells	% CD38+ of CD8+ T cells
ID42	T1	(as of viable CD45+CD3+CD8+CD4-)	CD45+CD3+CD8+CD4-HLA-DR+	T cytotoxic cells	% HLA-DR+ of CD8+ T cells
ID43	T1	(as of viable CD45+CD3+CD8+CD4-)	CD45+CD3+CD8+CD4-CD39+	T cytotoxic cells	% CD39+ of CD8+ T cells

Table S2. Annotation of cell subpopulations characterized in this study

ID	Tube	Parent Population	Markers	Category	Populations Name
ID44	T1	(as of viable CD45+CD3+CD8+CD4-)	CD45+CD3+CD8+CD4-CD103+	T cytotoxic cells	% CD103+ of CD8+ T cells
ID45	T1	(as of viable CD45+CD3+CD8+CD4-)	CD45+CD3+CD8+CD4-CD127+	T cytotoxic cells	% CD127+ of CD8+ T cells
ID46	T2	(as of viable CD45+CD3+CD8+CD4-)	CD45+CD3+CD8+CD4-IL17a+	T cytotoxic cells	% IL17A+ of CD8+ T cells (Tc17)
ID47	T2	(as of viable CD45+CD3+CD8+CD4-)	CD45+CD3+CD8+CD4-IL23r+	T cytotoxic cells	% IL23R+ of CD8+ T cells
ID48	T3	(as of viable CD45+CD3+CD8+CD4-)	CD45+CD3+CD8+CD4-IL2+	T cytotoxic cells	% IL2+ of CD8+ T cells
ID49	T3	(as of viable CD45+CD3+CD8+CD4-)	CD45+CD3+CD8+CD4-IFNg+	T cytotoxic cells	% INFg+ of CD8+ T cells
ID50	T3	(as of viable CD45+CD3+CD8+CD4-)	CD45+CD3+CD8+CD4-Perforin+	T cytotoxic cells	% Perforin+ of CD8+ T cells
ID51	T3	(as of viable CD45+CD3+CD8+CD4-)	CD45+CD3+CD8+CD4-TNFa+	T cytotoxic cells	% TNFa+ of CD8+ T cells
ID52	T1	(as of viable CD45+CD3+CD8+CD4-)	CD45+CD3+CD8+CD4-CD27+	T cytotoxic cells	% CD27+ of CD8+ T cells
ID53	T1	(as of viable CD45+CD3+CD8+CD4-)	CD45+CD3+CD8+CD4-CD45RA+	T cytotoxic cells	% CD45RA+ of CD8+ T cells
ID54	T1	(as of viable CD45+CD3+CD8+CD4-CD45RA+)	CD45+CD3+CD8+CD4-CD45RA+CD27+	T cytotoxic cells	% CD27+ of CD45RA+CD8+ T cells (Naïve CD8+ T)
ID55	T1	(as of viable CD45+CD3+CD8+CD4-CD45RA+)	CD45+CD3+CD8+CD4-CD45RA+CD27-	T cytotoxic cells	% CD27- of CD45RA+CD8+ T cells (EMRA CD8+ T)
ID56	T1	(as of viable CD45+CD3+CD8+CD4-)	CD45+CD3+CD8+CD4-CD45RA-	T cytotoxic cells	% CD45RA- of CD8+ T cells (CD45RA- memory CD8+ T)
ID57	T1	(as of viable CD45+CD3+CD8+CD4-CD45RA-)	CD45+CD3+CD8+CD4-CD45RA-CD27+CCR7+	T cytotoxic cells	% CD27+CCR7+ of CD45RA- memory CD8+ T cells (Central memory CD8+ T)
ID58	T1	(as of viable CD45+CD3+CD8+CD4-CD45RA-)	CD45+CD3+CD8+CD4-CD45RA-CD27-CCR7-	T cytotoxic cells	% CD27-CCR7- of CD45RA- memory CD8+ T cells (Effector memory CD8+ T)
ID59	T1	(as of viable CD45+CD3+CD4+CD8-)	CD45+CD3+CD4+CD8-CD25highFOXp3+	T regulatory cells	% CD25hi FoxP3+ of CD4+ T cells (Treg)
ID60	T1	(as of viable CD45+CD3+CD4+CD8-	CD45+CD3+CD4+CD8-CD25highFOXp3+CD38+	T regulatory cells	% CD38+ of Treg cells
ID61	T1	(as of viable CD45+CD3+CD4+CD8-	CD45+CD3+CD4+CD8-CD25highFOXp3+CD39+	T regulatory cells	% CD39+ of Treg cells
ID62	T1	(as of viable CD45+CD3+CD4+CD8-	CD45+CD3+CD4+CD8-CD25highFOXp3+CD103+	T regulatory cells	% CD103+ of Treg cells
ID63	T1	(as of viable CD45+CD3+CD4+CD8-	CD45+CD3+CD4+CD8-CD25highFOXp3+HLADR+	T regulatory cells	% HLA-DR+ of Treg cells
ID121	T1	(as of viable CD45+CD3+ CD4+CD8-)	CD45+CD3+CD4+CD8-CD45RA+CD27+	T helper cells	% CD45RA+CD27+ of CD4+ T cells
ID122	T1	(as of viable CD45+CD3+ CD4+CD8-)	CD45+CD3+CD4+CD8-CD45RA+CD27-	T helper cells	% CD45RA+CD27- of CD4+ T cells
ID123	T1	(as of viable CD45+CD3+ CD4+CD8-)	CD45+CD3+CD4+CD8-CD45RA-CD27-	T helper cells	% CD45RA-CD27- of CD4+ T cells
ID124	T1	(as of viable CD45+CD3+ CD4+CD8-)	CD45+CD3+CD4+CD8-CD45RA-CD27+	T helper cells	% CD45RA-CD27+ of CD4+ T cells
ID125	T1	(as of viable CD45+CD3+ CD4-CD8+)	CD45+CD3+CD4-CD8+CD45RA+CD27+	T cytotoxic cells	% CD45RA+CD27+ of CD8+ T cells
ID126	T1	(as of viable CD45+CD3+ CD4-CD8+)	CD45+CD3+CD4-CD8+CD45RA+CD27-	T cytotoxic cells	% CD45RA+CD27- of CD8+ T cells
ID127	T1	(as of viable CD45+CD3+ CD4-CD8+)	CD45+CD3+CD4-CD8+CD45RA-CD27-	T cytotoxic cells	% CD45RA-CD27- of CD8+ T cells
ID128	T1	(as of viable CD45+CD3+ CD4-CD8+)	CD45+CD3+CD4-CD8+CD45RA-CD27+	T cytotoxic cells	% CD45RA-CD27+ of CD8+ T cells
ID129	T2	(as of viable CD45+CD3+ CD4+CD8-)	CD45+CD3+CD4+CD8-CD146+	T helper cells	% CD146+ of CD4+ T cells
ID130	T2	(as of viable CD45+CD3+ CD4-CD8+)	CD45+CD3+CD4-CD8+CD146+	T cytotoxic cells	% CD146+ of CD8+ T cells
ID131	T3	(as of viable CD45+CD3+ CD4+CD8-)	CD45+CD3+CD4+CD8-CD45R0+	T helper cells	% CD45R0+ of CD4+ T cells
ID132	T3	(as of viable CD45+CD3+ CD4-CD8+)	CD45+CD3+CD4-CD8+CD45R0+	T cytotoxic cells	% CD45R0+ of CD8+ T cells

Table S2. Annotation of cell subpopulations characterized in this study

ID	Tube	Parent Population	Markers	Category	Populations Name
ID80	B2	(as of viable CD45+)	CD45+CD19+	B cells	% CD19+ of viable CD45+ (Total B cells)
ID81	B2	(as of viable CD45+CD19+CD20+)	CD45+CD19+CD20+CD80+	B cells	% CD80+ of CD20+ B cells (CD80+ activated mature B cells)
ID82	B2	(as of viable CD45+CD19+CD20+)	CD45+CD19+CD20+CD86+	B cells	% CD86+ of CD20+ B cells (CD86+ activated mature B cells)
ID83	B2	(as of viable CD45+CD19+CD20+)	CD45+CD19+CD20+IgA+	B cells	% IgA+ of CD20+ B cells (IgA+ mature B)
ID84	B2	(as of viable CD45+CD19+CD20+)	CD45+CD19+CD20+IgG+	B cells	% IgG+ of CD20+ B cells (IgG+ mature B)
ID85	B2	(as of viable CD45+CD19+CD20+)	CD45+CD19+CD20+IgM+IgD+	B cells	% IgM+IgD+ of CD20+ B cells (IgM+IgD+ mature B)
ID86	B2	(as of viable CD45+CD19+CD20+)	CD45+CD19+CD20+IgM-IgG-	B cells	% IgM-IgD- of CD20+ B cells (IgM-IgD- mature B)
ID87	B2	(as of viable CD45+CD19+)	CD45+CD19+CD20-CD27highCD38++	Plasmablast	% CD27hi CD38hi of CD20- B cells (Plasmablasts)
ID88	B2	(as of viable CD45+CD19+CD20-CD27highCD38++)	CD45+CD19+CD20-CD27highCD38++CD23+	Plasmablast	% CD23+ of plasmablasts
ID89	B2	(as of viable CD45+CD19+CD20-CD27highCD38++)	CD45+CD19+CD20-CD27highCD38++CD21+	Plasmablast	% CD21+ of plasmablasts
ID90	B2	(as of viable CD45+CD19+CD20+)	CD45+CD19+CD20+CD27-CD10+	Transitional B cells	% CD10+CD27- of CD20+ B cells (Transitional B)
ID91	B2	(as of viable CD45+CD19+CD20+CD27-CD10+)	CD45+CD19+CD20+CD27-CD10+CD38+	Transitional B cells	% CD38+ of transitional B cells
ID92	B2	(as of viable CD45+CD19+CD20+CD27-CD10+)	CD45+CD19+CD20+CD27-CD10+CD23+	Transitional B cells	% CD23+ of transitional B cells
ID93	B2	(as of viable CD45+CD19+CD20+CD27-CD10+)	CD45+CD19+CD20+CD27-CD10+CD21+	Transitional B cells	% CD21+ of transitional B cells
ID94	B2	(as of viable CD45+CD19+CD20+ (Minus viable CD45+CD19+CD20-CD27highCD38++) (minus viable CD45+CD19+CD27-CD10+))	CD45+CD19+CD20+IgD-CD27+	Mature B cells	% IgD-CD27+ of CD20+ B cells (IgD-CD27+ memory B, overlaps with CD20int plasmablasts)
ID95	B2	(as of viable CD45+CD19+CD20+IgD-CD27+ (Minus viable CD45+CD19+CD20-CD27highCD38++) (minus viable CD45+CD19+CD27-CD10+))	CD45+CD19+CD20+IgD-CD27+CD23+	Mature B cells	% CD23+ of IgD-CD27+ memory B cells
ID96	B2	(as of viable CD45+CD19+CD20+IgD-CD27+ (Minus viable CD45+CD19+CD20-CD27highCD38++) (minus viable CD45+CD19+CD27-CD10+))	CD45+CD19+CD20+IgD-CD27+CD38+	Mature B cells	% CD38+ of IgD-CD27+ memory B cells (overlaps with CD20int plasmablasts)
ID97	B2	(as of viable CD45+CD19+CD20+IgD-CD27+ (Minus viable CD45+CD19+CD20-CD27highCD38++) (minus viable CD45+CD19+CD27-CD10+))	CD45+CD19+CD20+IgD-CD27+CD80+	Mature B cells	% CD80+ of IgD-CD27+ memory B cells
ID98	B2	(as of viable CD45+CD19+CD20+IgD-CD27+ (Minus viable CD45+CD19+CD20-CD27highCD38++) (minus viable CD45+CD19+CD27-CD10+))	CD45+CD19+CD20+IgD-CD27+CD86+	Mature B cells	% CD86+ of IgD-CD27+ memory B cells

ID	Tube	Parent Population	Markers	Category	Populations Name
ID99	B2	(as of viable CD45+CD19+CD20+IgD-CD27+ (Minus viable CD45+CD19+CD20-CD27highCD38++) (minus viable CD45+CD19+CD27-CD10+))	CD45+CD19+CD20+IgD-CD27+IgA+	Mature B cells	% IgA+ of IgD-CD27+ memory B cells
ID100	B2	(as of viable CD45+CD19+CD20+IgD-CD27+ (Minus viable CD45+CD19+CD20-CD27highCD38++) (minus viable CD45+CD19+CD27-CD10+))	CD45+CD19+CD20+IgD-CD27+IgG+	Mature B cells	% IgG+ of IgD-CD27+ memory B cells
ID101	B2	(as of viable CD45+CD19+CD20+ (Minus viable CD45+CD19+CD20-CD27highCD38++) (minus viable CD45+CD19+CD27-CD10+))	CD45+CD19+CD20+IgD+CD27+	Mature B cells	% IgD+CD27+ of CD20+ B cells (IgD+CD27+ memory B, overlaps with CD20int plasmablasts)
ID102	B2	(as of viable CD45+CD19+CD20+IgD+CD27+ (Minus viable CD45+CD19+CD20-CD27highCD38++) (minus viable CD45+CD19+CD27-CD10+))	CD45+CD19+CD20+IgD+CD27+CD23+	Mature B cells	% CD23+ of IgD+CD27+ memory B cells
ID103	B2	(as of viable CD45+CD19+CD20+IgD+CD27+ (Minus viable CD45+CD19+CD20-CD27highCD38++) (minus viable CD45+CD19+CD27-CD10+))	CD45+CD19+CD20+IgD+CD27+CD38+	Mature B cells	% CD38+ of IgD+CD27+ memory B cells (overlaps with CD20int plasmablasts)
ID104	B2	(as of viable CD45+CD19+CD20+IgD+CD27+ (Minus viable CD45+CD19+CD20-CD27highCD38++) (minus viable CD45+CD19+CD27-CD10+))	CD45+CD19+CD20+IgD+CD27+CD80+	Mature B cells	% CD80+ of IgD+CD27+ memory B cells
ID105	B2	(as of viable CD45+CD19+CD20+IgD+CD27+ (Minus viable CD45+CD19+CD20-CD27highCD38++) (minus viable CD45+CD19+CD27-CD10+))	CD45+CD19+CD20+IgD+CD27+CD86+	Mature B cells	% CD86+ of IgD+CD27+ memory B cells
ID106	B2	(as of viable CD45+CD19+CD20+ (Minus viable CD45+CD19+CD20-CD27highCD38++) (minus viable CD45+CD19+CD27-CD10+))	CD45+CD19+CD20+IgD+CD27-	Mature B cells	% IgD+CD27- of CD20+ B cells (Naïve B, overlaps with CD20int plasmablasts)
ID107	B2	(as of viable CD45+CD19+CD20+IgD+CD27- (Minus viable CD45+CD19+CD20-CD27highCD38++) (minus viable CD45+CD19+CD27-CD10+))	CD45+CD19+CD20+IgD+CD27-CD21+	Mature B cells	% CD21+ of Naïve B cells

ID	Tube	Parent Population	Markers	Category	Populations Name
ID108	B2	(as of viable CD45+CD19+CD20+IgD+CD27- (Minus viable CD45+CD19+CD20-CD27highCD38++) (minus viable CD45+CD19+CD27-CD10+))	CD45+CD19+CD20+IgD+CD27-CD38+	Mature B cells	% CD38+ of Naïve B cells
ID109	B2	(as of viable CD45+CD19+CD20+IgD+CD27- (Minus viable CD45+CD19+CD20-CD27highCD38++) (minus viable CD45+CD19+CD27-CD10+))	CD45+CD19+CD20+IgD+CD27-CD80+	Mature B cells	% CD80+ of Naïve B cells
ID110	B2	(as of viable CD45+CD19+CD20+IgD+CD27- (Minus viable CD45+CD19+CD20-CD27highCD38++) (minus viable CD45+CD19+CD27-CD10+))	CD45+CD19+CD20+IgD+CD27-CD86+	Mature B cells	% CD86+ of Naïve B cells
ID111	B2	(as of viable CD45+CD19+CD20+IgD+CD27- (Minus viable CD45+CD19+CD20-CD27highCD38++) (minus viable CD45+CD19+CD27-CD10+))	CD45+CD19+CD20+IgD+CD27-IgA+	Mature B cells	% IgA+ of Naïve B cells
ID112	B2	(as of viable CD45+CD19+CD20+IgD+CD27- (Minus viable CD45+CD19+CD20-CD27highCD38++) (minus viable CD45+CD19+CD27-CD10+))	CD45+CD19+CD20+IgD+CD27-IgG+	Mature B cells	% IgG+ of Naïve B cells
ID113	B2	(as of viable CD45+CD19+CD20+ (Minus viable CD45+CD19+CD20-CD27highCD38++) (minus viable CD45+CD19+CD27-CD10+))	CD45+CD19+CD20+IgD-CD27-	Mature B cells	% IgD-CD27- of CD20+ B cells (IgD-CD27- memory B, overlaps with CD20int plasmablasts)
ID114	B2	(as of viable CD45+CD19+CD20+IgD-CD27- (Minus viable CD45+CD19+CD20-CD27highCD38++) (minus viable CD45+CD19+CD27-CD10+))	CD45+CD19+CD20+IgD-CD27-CD21+	Mature B cells	% CD21+ of IgD-CD27- memory B cells
ID115	B2	(as of viable CD45+CD19+CD20+IgD-CD27- (Minus viable CD45+CD19+CD20-CD27highCD38++) (minus viable CD45+CD19+CD27-CD10+))	CD45+CD19+CD20+IgD-CD27-CD38+	Mature B cells	% CD38+ of IgD-CD27- memory B cells
ID116	B2	(as of viable CD45+CD19+CD20+IgD-CD27- (Minus viable CD45+CD19+CD20-CD27highCD38++) (minus viable CD45+CD19+CD27-CD10+))	CD45+CD19+CD20+IgD-CD27-CD80+	Mature B cells	% CD80+ of IgD-CD27- memory B cells

ID	Tube	Parent Population	Markers	Category	Populations Name
ID117	B2	(as of viable CD45+CD19+CD20+IgD-CD27- (Minus viable CD45+CD19+CD20-CD27highCD38++) (minus viable CD45+CD19+CD27-CD10+))	CD45+CD19+CD20+IgD-CD27-CD86+	Mature B cells	% CD86+ of IgD-CD27- memory B cells
ID118	B2	(as of viable CD45+CD19+CD20+IgD-CD27- (Minus viable CD45+CD19+CD20-CD27highCD38++) (minus viable CD45+CD19+CD27-CD10+))	CD45+CD19+CD20+IgD-CD27-CD23+	Mature B cells	% CD23+ of IgD-CD27- memory B cells
ID119	B2	(as of viable CD45+CD19+CD20+IgD-CD27- (Minus viable CD45+CD19+CD20-CD27highCD38++) (minus viable CD45+CD19+CD27-CD10+))	CD45+CD19+CD20+IgD-CD27-IgA+	Mature B cells	% IgA+ of IgD-CD27- memory B cells
ID120	B2	(as of viable CD45+CD19+CD20+IgD-CD27- (Minus viable CD45+CD19+CD20-CD27highCD38++) (minus viable CD45+CD19+CD27-CD10+))	CD45+CD19+CD20+IgD-CD27-IgG+	Mature B cells	% IgG+ of IgD-CD27- memory B cells
ID133	NK2	(as of viable CD45+CD3-)	CD45+CD3-CD16+CD56+-	NK cells	% CD16+ and CD16-CD56+ of CD3- cells (total NK cells)
ID134	NK2	(as of viable CD45+CD3-CD16+CD56+-)	CD45+CD3-CD16+CD56+-CD5+	NK cells	% CD5+ of total NK cells (immature NK)
ID135	NK2	(as of viable CD45+CD3-CD16+CD56+-)	CD45+CD3-CD16+CD56+-CD335+	NK cells	% CD335(NKp46)+ of total NK cells
ID136	NK2	(as of viable CD45+CD3-CD16+CD56+-)	CD45+CD3-CD16+CD56+-CD158e+	NK cells	% CD158e(KIR3DL1)+ of total NK cells
ID137	NK2	(as of viable CD45+CD3-CD16+CD56+-)	CD45+CD3-CD16+CD56+-CD127+	NK cells	% CD127+ of total NK cells
ID138	NK2	(as of viable CD45+CD3-CD16+CD56+-)	CD45+CD3-CD16+CD56+-CD25+	NK cells	% CD25+ of total NK cells (activated NK cells)
ID139	NK2	(as of viable CD45+CD3-CD16+CD56+-)	CD45+CD3-CD16+CD56+-CD69+	NK cells	% CD69+ of total NK cells (activated NK cells)
ID140	NK2	(as of viable CD45+CD3-CD16+CD56+-)	CD45+CD3-CD16+CD56+-CD57+	NK cells	% CD57+ of total NK cells (mature NK cells)
ID141	NK2	(as of viable CD45+CD3-CD16+CD56+-)	CD45+CD3-CD16+CD56+-perforin+	NK cells	% Perforin+ of total NK cells
ID142	NK2	(as of viable CD45+CD3-CD16+CD56+-)	CD45+CD3-CD16+CD56+-IFNg+	NK cells	% IFNg+ of total NK cells
ID143	NK2	(as of viable CD45+CD3-CD16+CD56+-)	CD45+CD3-CD16+CD56+-CD8+	NK cells	% CD8+ of total NK cells

			50 mg									250 mg								
			4h vs 0h			24h vs 0h			24h vs 4h			4h vs 0h			24h vs 0h			24h vs 4h		
ID	Tube	Name	p_value	FDR	meandiff	p_value	FDR	meandiff	p_value	FDR	meandiff	p_value	FDR	meandiff	p_value	FDR	meandiff	p_value	FDR	meandiff
ID1	T1	% CD3+ of viable CD45+ cells (Total T cells)	0.014517	0.071924	-0.09193	0.417141	0.850433	0.018148	0.023989	0.121443	0.110076	0.017408	0.122582	-0.20647	0.076194	0.665209	0.029813	0.012509	0.102719	0.236287
ID2	T1	% CD4+ of total T cells	0.003929	0.042826	-0.17294	0.509178	0.850433	-0.0108	0.001998	0.032586	0.162137	0.000155	0.008622	-0.13481	0.571067	0.848188	-0.00431	1.39E-05	0.001519	0.1305
ID3	T1	% CD4+CD8+ of total T cells	0.517481	0.696364	0.045393	0.042276	0.834216	-0.05706	0.113713	0.23836	-0.10246	0.401408	0.568227	0.068449	0.339842	0.815455	-0.04778	0.089959	0.214418	-0.11623
ID4	T1	% CD8+ of total T cells	0.001261	0.042826	0.120415	0.514795	0.850433	0.006056	0.002231	0.032586	-0.11436	0.004356	0.059357	0.097194	0.801897	0.891523	0.003216	0.021987	0.131619	-0.09398
ID5	T1	% CD4-CD8- of total T cells	0.018392	0.081513	0.234606	0.656883	0.883954	0.032767	0.001527	0.032586	-0.20184	0.007175	0.072437	0.236441	0.313314	0.815455	-0.02381	0.006823	0.092965	-0.26025
ID6	T1, T2, T3	% CD4+ of total T cells	0.002055	0.042826	-0.17617	0.433129	0.850433	-0.01473	0.000894	0.032586	0.161438	0.001292	0.035197	-0.11857	0.513621	0.822402	-0.00797	3.53E-05	0.001537	0.110596
ID6	T1, T2, T3	% CD4+ of total T cells	0.003023	0.042826	-0.18345	0.339308	0.850433	-0.02157	0.00055	0.032586	0.161884	0.000158	0.008622	-0.12939	0.520603	0.822402	0.00443	4.23E-05	0.001537	0.133824
ID7	T3	% CD69+ of CD4+ T cells	0.073545	0.182192	0.340006	0.905715	0.939237	0.014523	0.056423	0.180887	-0.32548	0.066544	0.194843	0.271408	0.304296	0.815455	0.152134	0.577568	0.690066	-0.11927
ID11	T3	% CD40+ of CD4+ T cells	0.45292	0.641146	0.173716	0.505388	0.850433	-0.10638	0.079505	0.200542	-0.2801	0.119327	0.261682	0.181672	0.887954	0.91893	0.013953	0.115711	0.237972	-0.16772
ID12	T2	% CD161+ of CD4+ T cells	0.000518	0.042826	0.127418	0.111772	0.834216	0.04576	0.002737	0.032586	-0.08166	0.001216	0.035197	0.148243	0.486841	0.815455	0.03337	0.068286	0.206754	-0.11487
ID13	T2	% CD196+ of CD4+ T cells	0.012193	0.063288	0.157937	0.47113	0.850433	0.033327	0.130142	0.257918	-0.12461	0.002711	0.05401	0.250773	0.156007	0.683651	0.085892	0.061199	0.204766	-0.16488
ID14	T2	% IL17+ of CD4+ T cells	0.583617	0.743904	-0.0724	0.605985	0.883954	-0.13984	0.681576	0.816394	-0.06744	0.246221	0.406638	0.319136	0.340908	0.815455	0.210083	0.213331	0.381198	-0.10905
ID16	T2	% IL22+ of CD4+ T cells	0.547044	0.718408	-0.26063	0.299118	0.835997	0.187477	0.276268	0.46328	0.448102	0.003873	0.059357	0.625674	0.075181	0.665209	0.413017	0.379213	0.55857	-0.21266
ID17	T2	% IL23+ of CD4+ T cells	0.721827	0.833305	0.018469	0.613705	0.883954	0.060017	0.69011	0.81763	0.041547	0.183672	0.328201	0.309032	0.536523	0.831599	0.123566	0.001044	0.02845	-0.18547
ID18	T2	% CD161+ of CD4+ T cells	0.003764	0.042826	0.131666	0.283174	0.834216	0.041606	0.001747	0.032586	-0.09006	0.028311	0.147285	0.177628	0.468274	0.815455	0.051028	0.085186	0.211028	-0.1266
ID22	T2	% CD161- of CD4+ T cells	0.007589	0.051035	-0.01674	0.104765	0.834216	-0.00481	0.035653	0.141064	0.011928	0.030187	0.147285	-0.02046	0.669062	0.857293	-0.00225	0.147536	0.277265	0.018214
ID26	T3	% IL2+ of CD4+ T cells	0.608601	0.745365	0.124412	0.240847	0.834216	-0.21118	0.109207	0.237369	-0.33559	0.111031	0.260247	0.462515	0.393357	0.815455	0.178297	0.150644	0.278308	-0.28422
ID27	T3	% IFNg+ of CD4+ T cells	0.74335	0.844012	0.059971	0.729673	0.919052	0.058109	0.987645	0.99679	-0.00186	0.935988	0.95717	-0.00796	0.470089	0.815455	0.102769	0.490913	0.637018	0.11073
ID28	T3	% IL4+ of CD4+ T cells	0.948869	0.948869	0.010391	0.740134	0.919052	-0.03835	0.632181	0.776802	-0.04874	0.537701	0.705373	-0.0776	0.090914	0.665209	0.199678	0.21928	0.385509	0.277273
ID29	T3	% TNFa+ of CD4+ T cells	0.938559	0.948869	-0.00881	0.997661	0.997661	0.000336	0.907021	0.943626	0.009147	0.260756	0.424215	-0.14696	0.494392	0.815455	0.106099	0.239238	0.395106	0.253061
ID34	T1	% CD45RA+ of CD4+ T cells (Na?ve T)	0.018696	0.081513	-0.13062	0.857639	0.929824	-0.0062	0.07646	0.200542	0.124423	0.028588	0.147285	-0.11157	0.476514	0.815455	-0.02162	0.017465	0.111984	0.089957
ID35	T1	% CD45RA- of CD4+ T cells (Total memory CD4+ T)	0.042509	0.131408	0.083209	0.467156	0.850433	0.015425	0.034128	0.141064	-0.06778	0.073661	0.205873	0.062301	0.409277	0.815455	0.012939	0.033749	0.143483	-0.04936
ID36	T1	% CD27+CCR7- of memory CD4+ T cells (Effector memo	0.003918	0.042826	0.280158	0.216932	0.834216	0.04936	0.018982	0.121443	-0.2308	0.056786	0.193428	0.232474	0.818039	0.891523	-0.01183	0.08082	0.209748	-0.2443
ID37	T1	% CD27+CCR7+ of memory CD4+ T cells (Central memo	0.042733	0.131408	-0.04199	0.281644	0.834216	-0.00609	0.111062	0.237369	0.035901	0.103328	0.255971	-0.09439	0.549313	0.831599	-0.00599	0.093537	0.214418	0.088399
ID38	T1, T2, T3	% CD8+ of total T cells	0.008991	0.051035	0.104794	0.616567	0.883954	0.006958	0.008353	0.070035	-0.09784	0.019912	0.12767	0.070927	0.441063	0.815455	0.011828	0.015277	0.104077	-0.0591
ID38	T1, T2, T3	% CD8+ of total T cells	0.002238	0.042826	0.128844	0.050366	0.834216	0.02206	0.002384	0.032586	-0.10678	0.00731	0.072437	0.084766	0.038096	0.665209	-0.01918	0.006514	0.092965	-0.10395
ID39	T3	% CD69+ of CD8+ T cells	0.94051	0.948869	0.003638	0.249428	0.834216	-0.1093	0.069563	0.200542	-0.11294	0.0106	0.088876	0.296544	0.893639	0.91893	0.005127	0.002878	0.062742	-0.29142
ID46	T2	% IL17A+ of CD8+ T cells (Tc17)	0.815874	0.865967	-0.04967	0.478806	0.850433	0.151568	0.151462	0.289637	0.201238	0.197271	0.34131	0.182002	0.646777	0.852239	0.093497	0.720279	0.793034	-0.0885
ID47	T2	% IL23R+ of CD8+ T cells	0.265209	0.437997	-0.35211	0.446606	0.850433	-0.22595	0.309756	0.503931	0.126156	0.889905	0.923806	0.03517	0.646565	0.852239	-0.14311	0.552782	0.674401	-0.17828
ID48	T3	% IL2+ of CD8+ T cells	0.178396	0.342867	0.328603	0.156727	0.834216	0.210166	0.430593	0.60954	-0.11844	0.054858	0.193428	0.49041	0.029379	0.665209	0.369798	0.390492	0.560048	-0.12061
ID49	T3	% INFg+ of CD8+ T cells	0.726275	0.833305	-0.05301	0.845546	0.929824	-0.02266	0.610188	0.776802	0.03035	0.08991	0.233337	0.212662	0.624956	0.852239	0.065208	0.275181	0.435206	-0.14745
ID50	T3	% Perforin+ of CD8+ T cells	0.140218	0.315565	0.203033	0.226663	0.834216	-0.06097	0.028421	0.12908	-0.264	0.066694	0.194843	0.15739	0.853387	0.898429	-0.0087	0.062723	0.204766	-0.16609
ID51	T3	% TNFa+ of CD8+ T cells	0.332632	0.520848	-0.08678	0.19106	0.834216	-0.13253	0.100743	0.224101	-0.04576	0.943357	0.95717	0.009126	0.930109	0.938721	-0.00476	0.886593	0.911685	-0.01389
ID52	T1	% CD27+ of CD8+ T cells	0.043644	0.131408	-0.05778	0.792415	0.919052	0.004007	0.020319	0.121443	0.061784	0.1082	0.260247	-0.05262	0.091908	0.665209	0.006692	0.099545	0.217007	0.059315
ID53	T1	% CD45RA+ of CD8+ T cells	0.397056	0.584853	-0.05759	0.870111	0.929824	-0.00751	0.30536	0.503931	0.050088	0.043076	0.193428	-0.05266	0.408718	0.815455	0.026142	0.02415	0.131619	0.078798
ID54	T1	% CD27+ of CD45RA+CD8+ T cells (Na?ve CD8+ T)	0.028431	0.103811	-0.19797	0.557424	0.880569	0.025821	0.048132	0.16924	0.223789	0.12724	0.261682	-0.14307	0.415204	0.815455	-0.01694	0.094422	0.214418	0.126135
ID55	T1	% CD27- of CD45RA+CD8+ T cells (EMRA CD8+ T)	0.007238	0.051035	0.312147	0.680783	0.904943	0.032969	0.002989	0.032586	-0.27918	0.023777	0.141	0.315394	0.436622	0.815455	-0.05195	0.011555	0.102719	-0.36734
ID56	T1	% CD45RA- of CD8+ T cells (CD45RA- memory CD8+ T)	0.182426	0.342867	0.077784	0.645004	0.883954	0.014631	0.235174	0.406889	-0.06315	0.051632	0.193428	0.062076	0.389846	0.815455	-0.02433	0.074877	0.209617	-0.08641
ID57	T1	% CD27+CCR7+ of CD45RA- memory CD8+ T cells (Cent	0.332448	0.520848	-0.03523	0.435432	0.850433	-0.01369	0.504382	0.68722	0.021541	0.143322	0.289298	-0.08191	0.717207	0.881184	0.010368	0.028284	0.134041	0.092279
ID58	T1	% CD27-CCR7- of CD45RA- memory CD8+ T cells (Effect	0.020651	0.086576	0.133545	0.790727	0.919052	0.015331	0.075837	0.200542	-0.11821	0.084888	0.225678	0.231509	0.649736	0.852239	0.047386	0.013193	0.102719	-0.18412
ID59	T1	% CD25hi FoxP3+ of CD4+ T cells (Treg)	0.09864	0.238928	0.153351	0.93062	0.939237	0.004138	0.054814	0.180887	-0.14921	0.065649	0.194843	0.171738	0.82609	0.891523	-0.00767	0.102846	0.219809	-0.17941
ID60	T1	% CD38+ of Treg cells	0.040154	0.131408	0.175706	0.357936	0.850433	-0.05749	0.017641	0.120176	-0.23319	0.323324	0.496371	0.076118	0.122499	0.665209	-0.05676	0.140571	0.273611	-0.13288
ID61	T1	% CD39+ of Treg cells	0.044606	0.131408	0.083013	0.017387	0.834216	0.046895	0.218193	0.389886	-0.03612	0.814357	0.887649	0.010022	0.739407	0.88549	-0.01059	0.678641	0.775529	-0.02061
ID62	T1	% CD103+ of Treg cells	0.065962	0.175362	0.181605	0.105568	0.834216	-0.13221	0.007937	0.070035	-0.31381	0.017994	0.122582	0.180238	0.272656	0.815455	-0.08215	0.048262	0.17535	-0.26239
ID63	T1	% HLA-DR+ of Treg cells	0.144755	0.315565	0.123229	0.317748	0.850433	-0.07035	0.043309	0.157358	-0.19358	0.082893	0.225678	0.119867	0.135911	0.670907	-0.05659	0.076612	0.209617	-0.17646
ID80	B2	% CD19+ of viable CD45+ (Total B cells)	0.295831	0.481278	-0.06141	0.482385	0.850433	-0.06028	0.986845	0.99679	0.001133	0.839845	0.897481	-0.01021	0.116563	0.665209	-0.1252	0.027139	0.134041	-0.11499
ID81	B2	% CD80+ of CD20+ B cells (CD80+ activated mature B)	0.004678	0.046354	0.137877	0.818575	0.919052	-0.01165	0.012083	0.087803	-0.14953	0.375694	0.546008	0.116944	0.761069	0.891523	0.013874	0.285982	0.445315	-0.10307
ID82	B2	% CD86+ of CD20+ B cells (CD86+ activated mature B)	0.111979	0.265341	0.144877	0.708885	0.919052	-0.03439	0.075153	0.200542	-0.17927	0.28844	0.455652	0.125785	0.388378	0.815455	0.098186	0.838543	0.88739	-0.0276
ID83	B2	% IgA+ of CD20+ B cells (IgA+ mature B)	0.361603	0.555137	0.07311	0.651775	0.883954	-0.01624	0.088253	0.204836	-0.08935	0.948389	0.95717	0.002217	0.102331	0.665209	-0.03718	0.079007	0.209748	-0.0394
ID84	B2	% IgG+ of CD20+ B cells (IgG+ mature B)	0.031514	0.110806	-0.13016	0.151133	0.834216	-0.10951	0.727041	0.834183	0.020651	0.024578	0.141	-0.11027	0.106656	0.665209	-0.06552	0.275497	0.435206	0.044752
ID85	B2	% IgM+IgD+ of CD20+ B cells (IgM+IgD+ mature B)	0.759687	0.849346	-0.0086	0.268575	0.834216	0.025938	0.374926	0.567596	0.034543	0.8138	0.887649	-0.00278	0.800815	0.891523	0.005233	0.697265	0.775529	0.008008
ID86	B2	% IgM-IgD- of CD20+ B cells (IgM-IgD- mature B)	0.512706	0.696364	0.026844	0.649857	0.883954	-0.02324	0.462705	0.646601	-0.05008	0.120662	0.261682	-0.01999	0.30762	0.815455	-0.03282	0.608759	0.713492	-0.01283
ID87	B2	% CD27hi CD38hi of CD20- B cells (Plasmablasts)	0.17737	0.342867	0.260158	0.795299	0.919052	-0.03989	0.032015	0.139587	-0.30005	0.051912	0.193428	0.275701	0.485969	0.815455	-0.05694	0.030576	0.138868	-0.33264
ID88	B2	% CD23+ of plasmablasts	0.699381	0.819705	-0.23902	0.170605	0.834216	-0.13695	0.867299	0.919903	0.102065	0.212854	0.362516	-0.64532	0.489495	0.815455	-0.24463	0.52347	0.663467	0.400693
ID89	B2	% CD21+ of plasmablasts	0.154645	0.318043	-0.08196	0.390157	0.850433	-0.06315	0.63142	0.776802	0.018813	0.517366	0.696209	-0.04175	0.487142	0.815455	0.041304	0.416529	0.582073	0.083056
ID90	B2	% CD10+CD27- of CD20+ B cells (Transitional B)	0.919884	0.945919	0.008754	0.884585	0.936114	-0.0107	0.63427	0.776802	-0.01946	0.732085	0.831222	-0.01325	0.265722	0.815455	-0.03816	0.326301	0.493984	-0.02492
ID91	B2	% CD38+ of transitional B cells	0.600583	0.743904	-0.03835	0.172487	0.834216	-0.08671	0.323315	0.510744	-0.04836	0.393596	0.564499	-0.10695	0.501243	0.815455	-0.31338	0.631567	0.732349	-0.20642
ID92	B2	% CD23+ of transitional B cells	0.411951	0.598702	-0.41153	0.082875	0.834216	-0.82792	0.345421	0.530294	-0.41639	0.095449	0.241952	0.763171	0.260195	0.815455	0.448187	0.553248	0.674401	-0.31498
ID93	B2	% CD21+ of transitional B cells	0.153944	0.318043	-0.00498	0.924018	0.939237	-8.5E-05	0.15089	0.289637	0.004897	0.125404	0.261682	-0.00467	0.857217	0.898429	-0.00047	0.006	0.092965	0.004197
ID94	B2	% IgD-CD27+ of CD20+ B cells* (IgD-CD27+ memory B)	0.832758	0.872795	-0.01603	0.264735	0.834216	-0.06646	0.323074	0.510744	-0.05043	0.181721	0.328201	-0.05443	0.07537	0.665209	-0.06233	0.554727	0.674401	-0.00791
ID95	B2	% CD23+ of IgD-CD27+ memory B cells	0.217819	0.382939	0.127629	0.210652	0.834216	0.153133	0.644823	0.780952	0.025505	0.53	0.704512	0.064856	0.360723	0.815455	-0.31154	0.253347	0.412161	-0.3764

Table S3. Coherent changes in frequencies of characterized cell populations.

			50 mg									250 mg								
			4h vs 0h			24h vs 0h			24h vs 4h			4h vs 0h			24h vs 0h			24h vs 4h		
ID	Tube	Name	p_value	FDR	meandiff	p_value	FDR	meandiff	p_value	FDR	meandiff	p_value	FDR	meandiff	p_value	FDR	meandiff	p_value	FDR	meandiff
ID96	B2	% CD38+ of IgD-CD27+ memory B cells**	0.264812	0.437997	0.153752	0.543051	0.870479	0.045144	0.120486	0.243203	-0.10861	0.017025	0.122582	0.281592	0.960323	0.960323	-0.0044	0.022943	0.131619	-0.286
ID97	B2	% CD80+ of IgD-CD27+ memory B cells	0.184846	0.342867	0.075276	0.428447	0.850433	0.068238	0.917655	0.943626	-0.00704	0.180274	0.328201	0.164098	0.638024	0.852239	-0.02236	0.107769	0.2259	-0.18646
ID98	B2	% CD86+ of IgD-CD27+ memory B cells	0.587934	0.743904	0.021611	0.926882	0.939237	-0.00542	0.761081	0.855235	-0.02703	0.411609	0.575197	0.054942	0.12816	0.665209	-0.16026	0.159764	0.290237	-0.2152
ID99	B2	% IgA+ of IgD-CD27+ memory B cells	0.597321	0.743904	0.013049	0.780811	0.919052	0.006305	0.796282	0.875151	-0.00674	0.375615	0.546008	0.03022	0.467816	0.815455	-0.02411	0.073914	0.209617	-0.05433
ID100	B2	% IgG+ of IgD-CD27+ memory B cells	0.053676	0.153966	-0.14423	0.464756	0.850433	-0.04029	0.02165	0.121443	0.103936	0.550061	0.705373	-0.02639	0.348688	0.815455	-0.05	0.7847	0.843468	-0.02361
ID101	B2	% IgD+CD27+ of CD20+ B cells* (IgD+CD27+ memory B	0.887607	0.921421	-0.02059	0.801479	0.919052	-0.02786	0.869266	0.919903	-0.00727	0.112216	0.260247	-0.07558	0.011336	0.665209	-0.14035	0.231124	0.392191	-0.06477
ID102	B2	% CD23+ of IgD+CD27+ memory B cells	0.006389	0.049744	-0.19551	0.108904	0.834216	-0.27605	0.516897	0.693995	-0.08054	0.599074	0.742034	-0.04531	0.608344	0.852239	-0.22738	0.690748	0.775529	-0.18208
ID103	B2	% CD38+ of IgD+CD27+ memory B cells***	0.662972	0.79411	0.033775	0.740365	0.919052	0.033709	0.999395	0.999395	-6.6E-05	0.294393	0.458411	0.5967	0.204585	0.796419	0.56675	0.860135	0.892902	-0.02995
ID104	B2	% CD80+ of IgD+CD27+ memory B cells	0.33449	0.520848	0.146663	0.653246	0.883954	0.089971	0.603552	0.776802	-0.05669	0.159983	0.314816	0.307144	0.300164	0.815455	-0.12054	0.034225	0.143483	-0.42768
ID105	B2	% CD86+ of IgD+CD27+ memory B cells	0.1438	0.315565	0.130523	0.637607	0.883954	0.058328	0.409394	0.59553	-0.0722	0.050325	0.193428	0.142777	0.193423	0.780857	-0.26368	0.098587	0.217007	-0.40646
ID106	B2	% IgD+CD27- of CD20+ B cells* (Na?ve B)	0.763632	0.849346	-0.00852	0.39428	0.850433	0.019827	0.251307	0.428008	0.028351	0.785622	0.882681	0.004071	0.575834	0.848188	0.007037	0.38824	0.560048	0.002966
ID107	B2	% CD21+ of Na?ve B cells	0.479321	0.66982	0.031421	0.190226	0.834216	-0.03071	0.082792	0.200542	-0.06213	0.851841	0.900876	-0.00648	0.839283	0.896881	-0.00667	0.984576	0.984576	-0.0002
ID108	B2	% CD38+ of Na?ve B cells	0.385599	0.583131	0.073453	0.825843	0.919052	0.019514	0.224802	0.395217	-0.05394	0.19248	0.338393	-0.09238	0.020363	0.665209	0.083197	0.036495	0.14733	0.175579
ID109	B2	% CD80+ of Na?ve B cells	0.067698	0.175693	0.197671	0.384386	0.850433	0.118458	0.16935	0.318262	-0.07921	0.632119	0.765375	0.02947	0.386599	0.815455	-0.13526	0.439551	0.598889	-0.16473
ID110	B2	% CD86+ of Na?ve B cells	0.805477	0.865967	0.024034	0.014117	0.834216	-0.17418	0.07725	0.200542	-0.19821	0.859551	0.900876	0.015773	0.100936	0.665209	-0.20832	0.144609	0.276534	-0.22409
ID111	B2	% IgA+ of Na?ve B cells	0.191688	0.342867	0.061835	0.276523	0.834216	-0.04672	0.089458	0.204836	-0.10856	0.275511	0.441628	-0.03248	0.120982	0.665209	-0.0415	0.556845	0.674401	-0.00902
ID112	B2	% IgG+ of Na?ve B cells	0.43305	0.621085	-0.16104	0.09308	0.834216	-0.21889	0.628554	0.776802	-0.05785	0.423527	0.577055	-0.10047	0.062792	0.665209	-0.34448	0.06575	0.204766	-0.24401
ID113	B2	% IgD-CD27- of CD20+ B cells* (IgD-CD27- memory B)	0.190406	0.342867	0.066725	0.514941	0.850433	0.025947	0.214098	0.388945	-0.04078	0.231646	0.388453	0.049552	0.31052	0.815455	0.065508	0.582441	0.690066	0.015956
ID114	B2	% CD21+ of IgD-CD27- memory B cells	0.570591	0.74041	-0.00191	0.645759	0.883954	0.002068	0.404677	0.59553	0.003976	0.621695	0.761401	0.003841	0.09286	0.665209	-0.01286	0.137405	0.272311	-0.0167
ID115	B2	% CD38+ of IgD-CD27- memory B cells	0.533508	0.709176	-0.04669	0.280691	0.834216	-0.07959	0.79038	0.875151	-0.0329	0.834879	0.897481	0.014779	0.656771	0.852239	0.033144	0.849602	0.890448	0.018365
ID116	B2	% CD80+ of IgD-CD27- memory B cells	0.791513	0.86275	-0.02325	0.275657	0.834216	-0.13381	0.052911	0.180227	-0.11056	0.336766	0.509826	0.277275	0.676396	0.857293	0.063856	0.233875	0.392191	-0.21342
ID117	B2	% CD86+ of IgD-CD27- memory B cells	0.818299	0.865967	0.011488	0.587721	0.883954	-0.01803	0.341233	0.530294	-0.02952	0.645035	0.765375	0.034428	0.156801	0.683651	-0.20337	0.226334	0.391594	-0.23779
ID118	B2	% CD23+ of IgD-CD27- memory B cells	0.390537	0.583131	-0.46875	0.810412	0.919052	-0.02329	0.475632	0.656251	0.445457	0.566583	0.714722	-0.05632	0.54385	0.831599	-0.03091	0.7775	0.843468	0.025413
ID119	B2	% IgA+ of IgD-CD27- memory B cells	0.627728	0.760248	0.009569	0.473068	0.850433	-0.07718	0.420772	0.603476	-0.08675	0.646005	0.765375	-0.01844	0.400881	0.815455	-0.06697	0.346767	0.517776	-0.04853
ID120	B2	% IgG+ of IgD-CD27- memory B cells	0.002346	0.042826	-0.10617	0.223817	0.834216	-0.12869	0.809898	0.875151	-0.02252	0.009739	0.088462	-0.11589	0.141567	0.670907	-0.09835	0.7893	0.843468	0.017534
ID121	T1	% CD45RA+CD27+ of CD4+ T cells	0.009364	0.051035	-0.18775	0.293027	0.835997	-0.04167	0.080489	0.200542	0.146081	0.126327	0.261682	-0.10535	0.747386	0.88549	-0.01354	0.026153	0.134041	0.091815
ID122	T1	% CD45RA+CD27- of CD4+ T cells	0.152179	0.318043	0.27609	0.062656	0.834216	-0.13135	0.025625	0.121443	-0.40744	0.171839	0.322939	0.318367	0.590892	0.852239	-0.07054	0.040291	0.156847	-0.3889
ID123	T1	% CD45RA-CD27- of CD4+ T cells	0.009216	0.051035	0.290867	0.728502	0.919052	0.014384	0.005578	0.05527	-0.27648	0.046631	0.193428	0.259226	0.800393	0.891523	0.009952	0.076924	0.209617	-0.24927
ID124	T1	% CD45RA-CD27+ of CD4+ T cells	0.026453	0.102979	0.050385	0.127046	0.834216	0.030309	0.187278	0.345987	-0.02008	0.361357	0.53956	-0.0435	0.457535	0.815455	-0.01109	0.408052	0.577632	0.032403
ID125	T1	% CD45RA+CD27+ of CD8+ T cells	0.056382	0.157581	-0.17688	0.778995	0.919052	-0.0199	0.090203	0.204836	0.156975	0.059983	0.194843	-0.23877	0.74149	0.88549	-0.0159	0.048148	0.17535	0.222872
ID126	T1	% CD45RA+CD27- of CD8+ T cells	0.028572	0.103811	0.175934	0.493222	0.850433	0.026387	0.081756	0.200542	-0.14955	0.167695	0.32068	0.155941	0.321841	0.815455	-0.05784	0.08343	0.211028	-0.21379
ID127	T1	% CD45RA-CD27- of CD8+ T cells	0.058231	0.158678	0.222658	0.157192	0.834216	0.065754	0.073734	0.200542	-0.1569	0.061523	0.194843	0.190335	0.719499	0.881184	-0.01916	0.008621	0.102719	-0.2095
ID128	T1	% CD45RA-CD27+ of CD8+ T cells	0.236957	0.409973	0.073227	0.425593	0.850433	0.021244	0.409768	0.59553	-0.05198	0.420475	0.577055	0.052476	0.32478	0.815455	-0.03955	0.299818	0.460283	-0.09203
ID129	T2	% CD146+ of CD4+ T cells	0.017299	0.081513	0.183934	0.038367	0.834216	0.177543	0.912885	0.943626	-0.00639	0.126351	0.261682	0.457331	0.053425	0.665209	0.222563	0.451661	0.607791	-0.23477
ID130	T2	% CD146+ of CD8+ T cells	0.692707	0.819705	-0.07858	0.764933	0.919052	-0.02761	0.755448	0.855235	0.050968	0.975558	0.975558	-0.00277	0.386121	0.815455	0.087059	0.133562	0.269597	0.08983
ID131	T3	% CD45R0+ of CD4+ T cells	0.035744	0.121752	0.088504	0.493525	0.850433	0.017346	0.036237	0.141064	-0.07116	0.007253	0.072437	0.090925	0.090172	0.665209	0.038762	0.062547	0.204766	-0.05216
ID132	T3	% CD45R0+ of CD8+ T cells	0.489666	0.675615	0.050976	0.39163	0.850433	-0.13709	0.118357	0.243203	-0.18807	0.055195	0.193428	0.204063	0.634806	0.852239	0.036078	0.090588	0.214418	-0.16799
ID133	NK2	% CD16+ and CD16-CD56+ of CD3- cells (total NK cells)	0.006112	0.049744	0.263934	0.186369	0.834216	-0.04281	0.002395	0.032586	-0.30674	0.002973	0.05401	0.24952	0.597451	0.852239	0.033306	0.010773	0.102719	-0.21621
ID134	NK2	% CD5+ of total NK cells (immature NK)	0.006021	0.049744	-0.34107	0.86335	0.929824	-0.01386	0.01044	0.08128	0.327212	0.031079	0.147285	-0.32337	0.178114	0.746707	0.072822	0.010741	0.102719	0.396192
ID135	NK2	% CD335(NKp46)+ of total NK cells	0.026365	0.102979	-0.30167	0.237864	0.834216	-0.08055	0.041007	0.154131	0.221117	0.16174	0.314816	0.159424	0.817101	0.891523	0.048324	0.426178	0.588017	-0.1111
ID136	NK2	% CD158e(KIR3DL1)+ of total NK cells	0.122534	0.284175	-0.26961	0.641392	0.883954	-0.11317	0.620016	0.776802	0.156441	0.793603	0.882681	0.012086	0.032512	0.665209	-0.09889	0.064762	0.204766	-0.11098
ID137	NK2	% CD127+ of total NK cells	0.070099	0.177692	0.077656	0.529071	0.860728	-0.01951	0.07582	0.200542	-0.09717	0.067927	0.194843	0.150295	0.821193	0.891523	0.039952	0.482152	0.637018	-0.11034
ID138	NK2	% CD25+ of total NK cells (activated NK cells)	0.243549	0.414794	-0.09449	0.100336	0.834216	-0.0599	0.700234	0.820704	0.034591	0.549243	0.705373	-0.03064	0.244036	0.815455	-0.07015	0.490905	0.637018	-0.0395
ID139	NK2	% CD69+ of total NK cells (activated NK cells)	0.782721	0.861784	0.026552	0.826304	0.919052	-0.02017	0.718508	0.833163	-0.04672	0.697621	0.800428	0.049822	0.927312	0.938721	0.013899	0.690533	0.775529	-0.03592
ID140	NK2	% CD57+ of total NK cells (mature NK cells)	0.002653	0.042826	0.072035	0.497179	0.850433	-0.01015	0.025124	0.121443	-0.08219	0.046404	0.193428	0.054451	0.379004	0.815455	-0.00723	0.015123	0.104077	-0.06168
ID141	NK2	% Perforin+ of total NK cells	0.008796	0.051035	0.060647	0.423547	0.850433	-0.01785	0.02557	0.121443	-0.0785	0.570466	0.714722	-0.01816	0.362389	0.815455	-0.05594	0.506357	0.649328	-0.03778
ID142	NK2	% IFNg+ of total NK cells	0.19188	0.342867	0.041063	0.907912	0.939237	-0.01151	0.522088	0.693995	-0.05258	0.692869	0.800428	0.076131	0.817577	0.891523	0.0543	0.895865	0.912611	-0.02183
ID143	NK2	% CD8+ of total NK cells	0.176703	0.342867	0.026493	0.216582	0.834216	0.022681	0.81092	0.875151	-0.00381	0.679861	0.796827	0.008842	0.707899	0.881184	0.007911	0.959133	0.968014	-0.00093

Table S3. Coherent changes in frequencies of characterized cell populations.
